# Multiplexed Surface Electrode Arrays Based on Metal Oxide Thin‐Film Electronics for High‐Resolution Cortical Mapping

**DOI:** 10.1002/advs.202308507

**Published:** 2023-12-25

**Authors:** Horacio Londoño‐Ramírez, Xiaohua Huang, Jordi Cools, Anna Chrzanowska, Clément Brunner, Marco Ballini, Luis Hoffman, Soeren Steudel, Cédric Rolin, Carolina Mora Lopez, Jan Genoe, Sebastian Haesler

**Affiliations:** ^1^ Department of Neuroscience, Leuven Brain Institute Katholieke Universiteit (KU) Leuven Leuven 3001 Belgium; ^2^ Neuroelectronics Research Flanders (NERF) Leuven 3001 Belgium; ^3^ imec Leuven 3001 Belgium; ^4^ Flanders Institute for Biotechnology (VIB) Gent 9052 Belgium; ^5^ Department of Electrical Engineering (ESAT) Katholieke Universiteit (KU) Leuven Leuven 3001 Belgium; ^6^ Department of Biology Katholieke Universiteit (KU) Leuven Leuven 3001 Belgium; ^7^ Present address: Thermo Fisher Scientific 3001 Leuven Belgium; ^8^ Present address: Microphone Business Unit, TDK InvenSense 20057 Milan Italy; ^9^ Present address: Swave Photonics 3001 Leuven Belgium; ^10^ Present address: MICLEDI Microdisplays 3001 Leuven Belgium

**Keywords:** µECoGs, a‐IGZO, electrocorticography, electrode arrays, flexible electronics, thin‐film transistors, time‐division multiplexing

## Abstract

Electrode grids are used in neuroscience research and clinical practice to record electrical activity from the surface of the brain. However, existing passive electrocorticography (ECoG) technologies are unable to offer both high spatial resolution and wide cortical coverage, while ensuring a compact acquisition system. The electrode count and density are restricted by the fact that each electrode must be individually wired. This work presents an active micro‐electrocorticography (µECoG) implant that tackles this limitation by incorporating metal oxide thin‐film transistors (TFTs) into a flexible electrode array, allowing to address multiple electrodes through a single shared readout line. By combining the array with an incremental‐ΔΣ readout integrated circuit (ROIC), the system is capable of recording from up to 256 electrodes virtually simultaneously, thanks to the implemented 16:1 time‐division multiplexing scheme, offering lower noise levels than existing active µECoG arrays. In vivo validation is demonstrated acutely in mice by recording spontaneous activity and somatosensory evoked potentials over a cortical surface of ≈8×8 mm^2^. The proposed neural interface overcomes the wiring bottleneck limiting ECoG arrays, holding promise as a powerful tool for improved mapping of the cerebral cortex and as an enabling technology for future brain‐machine interfaces.

## Graphical abstract

Increasing electrode count of flexible electrode arrays in a sustainable way: Incorporating metal oxide thin‐film transistors into flexible micro‐electrocorticography (µECoG) electrode arrays enables multiplexing of the recording electrodes, overcoming the wiring bottleneck faced by current µECoG technologies. By combining the arrays with a dedicated silicon readout chip, 256 electrodes can be recorded simultaneously in time‐division multiplexing mode employing only 16 channels.

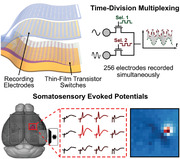

## 1 Introduction

Neurological disorders pose an enormous social and economic burden on affected individuals and health care systems throughout the world.^[^ ^1^ ^]^ This calls for the development of novel diagnostics, treatments, and cures.^[^ ^2^ ^]^ A key approach in diagnostic and therapeutic applications in the clinic involves direct physical interfacing with the brain to either record electrical activity in vivo,^[^ ^3^ ^]^ or to electrically modulate brain activity.^[^ ^4^ ^]^

An established application in clinical practice today involves placing grid electrodes on the surface of the brain to perform electrocorticography (ECoG). ECoG recordings are used for brain mapping in conditions such as focal epilepsy, in which electrode grids are used to delineate the brain region from which seizures originate.^[^ ^5^ ^]^ Additionally, electrical stimulation from ECoG grids is used for intraoperative functional mapping of cortical regions.^[^ ^6^ ^]^ More recently, ECoG electrode arrays have been employed in the development of brain‐computer interfaces, such as speech prostheses for paralyzed patients unable to communicate,^[^ ^7^ ^]^ and visual prostheses for blind individuals.^[^ ^8^ ^]^ ECoG arrays have also been used as part of a brain‐spine interface, enabling a paralyzed individual with chronic tetraplegia to stand and walk naturally.^[^ ^9^ ^]^ Advanced ECoG arrays will play a central role in many future neurotechnology applications, given their relatively favorable compromise between invasiveness, spatial resolution, brain coverage and signal stability across long time scales compared to brain‐penetrating probes on one hand and non‐invasive electroencephalography (EEG) electrodes on the other.^[^ ^5^ ^]^

Clinical ECoG grids are based on passive technologies, offering limited electrode count, reduced conformability to the brain curvature, and low spatial resolution, having electrode sizes in the order of millimeters.^[^ ^5^ ^]^ Innovations in ECoG technologies, such as the incorporation of microfabrication techniques borrowed from the semiconductor industry and the use of thin and flexible substrates, have empowered the development of novel devices with higher electrode count, spatial resolution, and improved biocompatibility.^[^ ^10^, ^11^, ^12^, ^13^ ^]^

These micro‐ECoG (µECoG) arrays with micro‐scale spatial resolution (<1 mm) have become an enabling technology for neuroscience research, providing access to localized information not available with standard methods.^[^ ^12^, ^13^, ^14^, ^15^, ^16^, ^17^, ^18^ ^]^ For instance, µECoG arrays have revealed the possibility of capturing action potentials from the surface of the human cortex with non‐penetrating electrodes,^[^ ^12^ ^]^ as well as other novel cortical signals not locked to rhythmic brain activity.^[^ ^14^ ^]^ Furthermore, their high‐resolution and broad‐area coverage allows for the exploration of neural processing and functional interactions across local and broadly distributed networks during different stages of neural computation.^[^ ^5^ ^]^

Moreover, the use of advanced µECoG arrays in diagnostic and therapeutic applications is promising. In the context of drug‐resistant epilepsy, high‐channel count arrays could significantly improve the delineation of functional and pathological tissue boundaries for surgical recession by offering more refined biomarkers, such as microscale seizures and high‐frequency oscillations, not detectable with clinical ECoG grids.^[^ ^15^, ^16^, ^17^ ^]^ Additionally, these arrays hold the potential to significantly enhance the performance of future brain‐computer interface systems. For example, advanced µECoG arrays have been instrumental in the development of high‐performance speech neuroprostheses, as their broad cortical coverage and high spatial density have proven essential in improving speech decoding from articulatory vocal‐tract representations distributed throughout the sensorimotor cortex.^[^ ^18^ ^]^

However, increasing the electrode count of passive µECoG arrays to offer high‐resolution recordings covering a large area of the cortical surface while ensuring a compact acquisition system is challenging and has not been reported to date.^[^ ^13^ ^]^ Too many metal lines need to be individually wired out, causing connectors to become bulky and ultimately precluding having a fully implantable system with high channel count.

Active µECoG arrays have tackled these limitations by incorporating transistors into flexible electrode arrays, enabling the multiplexing of multiple electrodes into few readout data lines. For a multiplexed array organized in a matrix configuration of *N* × *M* elements, with *N* being the multiplexing ratio, only *N* + *M* interconnects and *M* recording channels are necessary. *N* additional lines are needed to select the rows connected to the *M* recording channels. In contrast, for an equivalent passive array, *N* × *M* connections and channels would be required. As a result, current brain interfaces based on active µECoG arrays can achieve high spatial resolution and large cortical coverage, capable of recording from up to ≈1000 electrodes employing few connecting wires.^[^ ^19^, ^20^, ^21^, ^22^ ^]^ However, these technologies (based on silicon nanomembranes or on graphene transistors) suffer from relatively high noise levels (in the tens of microvolts), utilize fabrication flows difficult to transfer outside academic labs, and a further increase in channel count is challenging without degrading the electrical performance of the devices. In conclusion, low‐noise, high‐resolution multiplexed recordings have not yet been achieved in active µECoG arrays.

Here, we present a flexible, active µECoG array for high‐density neural recordings that capitalizes on advances in thin‐film electronics.^[^ ^23^, ^24^ ^]^ By incorporating metal oxide thin‐film transistors (TFTs) on each recording pixel, our array overcomes the wiring bottleneck limiting passive ECoG arrays, allowing us to address hundreds of closely spaced electrodes with a reduced number of lines – an indispensable step for a miniaturized and convenient connection scheme. The arrays can be operated in addressing mode, where subsets of electrodes can be selected and read employing standard electrophysiology acquisition systems. Furthermore, by combining the arrays with a dedicated incremental‐ΔΣ readout integrated circuit (ROIC) based on complementary metal‐oxide‐semiconductor (CMOS) silicon electronics,^[^ ^25^, ^26^ ^]^ the arrays can be operated in a time‐division multiplexing fashion, allowing for the virtually simultaneous recording of up to 256 electrodes (300‐µm electrode diameter, 500‐µm electrode pitch), employing only 16 recording channels. We characterized the arrays in vitro and validated them in vivo by recording acutely spontaneous activity and somatosensory evoked potentials from anesthetized mice over a cortical surface of 8×8 mm^2^. This work emphasizes the µECoG array's fabrication, characterization, and presents a comprehensive in vivo validation of the complete implant system, complementing our previous work focused on the design of the ROIC.^[^ ^25^, ^26^ ^]^ Compared to existing active µECoG arrays, our system offers (1) lower noise levels over a broader frequency range (2.65 µV_rms_ over 1 to 500 Hz), (2) a compact readout circuit (1.25 × 1.25 mm^2^ CMOS ROIC),^[^ ^25^, ^26^ ^]^ and (3) a scalable fabrication process compatible with thin‐film semiconductor foundries.^[^ ^24^, ^27^ ^]^

## 2 Results and Discussion

### 2.1 Multiplexed µECoG Arrays Based on Metal Oxide Thin‐Film Transistors

Our approach for increasing the number of recording electrodes without linearly scaling the number of required readout wires relies on integrating amorphous indium‐gallium‐zinc oxide (a‐IGZO) thin‐film transistors into flexible µECoG arrays. Incorporating transistors into the arrays enables multiplexing the recorded signals, either by addressing a subset of electrodes through channel‐selection multiplexing, or by recording from all electrodes virtually simultaneously employing time‐division multiplexing.

**Figure 1:**
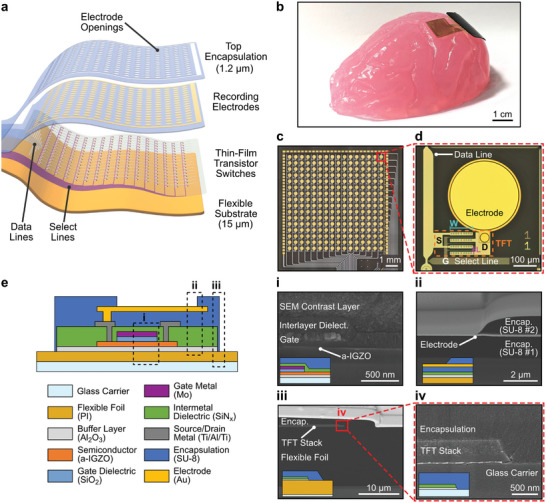
µECoG array based on a‐IGZO thin‐film transistors. a) Exploded‐view illustration highlighting the key elements of the flexible µECoG array. b) Photograph of a released flexible µECoG array conforming to the curvature of a macaque monkey brain phantom made of agarose gel. c) Microphotograph of a 256‐channel µECoG array (16×16 pixels, 500‐µm electrode pitch and ≈1×1 cm^2^ array dimensions) (© 2022 IEEE. Reprinted, with permission, from^[^ ^26^ ^]^) and d) magnification of a single pixel, consisting of an a‐IGZO select transistor (W/L  =  300 µm/3 µm) and a gold electrode (300 µm in diameter). Transistor's gate (G), source (S), and drain (D), as well as channel width (W) and length (L) are indicated in the figure. The TFT has interdigitated source and drain contacts, with the channel width divided into three sections. e) Schematic cross‐section of the flexible µECoG array stack, including the thin‐film transistor and the recording electrode. i) SEM cross‐section of a self‐aligned a‐IGZO TFT gate stack. ii) FIB/SEM cross‐section of a gold electrode. iii) FIB/SEM cross‐section of the lateral encapsulation protecting the edges of the µECoG array. iv) SEM cross‐section of the region highlighted with the dotted red box in (iii), emphasizing the lateral encapsulation. i) – iv) include insets with a schematic cross‐section of the layers present in the SEM image.

The active µECoG arrays can be divided in four key elements: a flexible substrate (15‐µm‐thick polyimide foil), a TFT backplane, recording gold electrodes, and a top encapsulation (1.2‐µm‐thick SU‐8 layer) (**Figure** **1**a). Arrays follow an *active‐matrix* architecture, in which each recording pixel consists of a sensing electrode and a pass transistor acting as a switch, allowing pixel selection by turning ON or OFF the corresponding switch. *Select lines* are used to activate different rows of pixels, which are then read simultaneously through the *data lines*. By switching between the selected rows, multiple electrodes can be addressed sequentially using a single data line. It is worth clarifying that our sensing pixel corresponds to a *passive‐pixel sensor*, as it consists of a passive metal electrode that is read out through the switch transistor, without any in‐pixel amplification. This simple implementation minimizes the impact of the transistor on the overall system's noise, since in this case the main contribution of the TFT comes from the thermal noise of its ON resistance.^[^ ^28^ ^]^

At their core, our µECoG arrays have the metal oxide thin‐film transistor. Their fabrication is built upon the process flow for self‐aligned (SA) top‐gate a‐IGZO TFTs with SiO_2_ gate dielectric previously developed in our group.^[^ ^27^ ^]^ The use of metal oxide TFTs as switching elements offers the advantage of providing very low currents in the off state, minimizing leakage and crosstalk between pixels in the array. Fabrication of the µECoG arrays required additional postprocessing steps over the TFT backplane, including the incorporation of recording electrodes and a suitable encapsulation strategy. Gold was chosen as the electrode material due to its proven biocompatibility and its ease of integration into our existing process flow through wet‐etch patterning. Compared to alternative multiplexed µECoG technologies, our approach has the major advantage that it is based on an established process developed for flexible active‐matrix organic light‐emitting diode (AMOLED) displays, which allows it to be transferable to semiconductor foundries.^[^ ^29^ ^]^

The fabrication follows standard microfabrication process steps (for details, see Experimental Section: *Fabrication of µECoG Electrode Arrays*). Schematic cross‐sections, along with micrographs of the main process steps of the fabrication of the µECoG array are presented in Figure S1 (Supporting Information). The total thickness of the array was ≈18.5 µm. A photograph of a free‐standing flexible µECoG array conforming to the curvature of a macaque monkey brain phantom made of agarose gel, as well as microphotographs of a 16 × 16 array and of a single recording pixel formed by a gold electrode and an a‐IGZO TFT are displayed in Figure 1b–d, respectively.

Figure 1e presents a schematic cross‐section of the finalized µECoG array stack, including the two main components of the recording pixel: the SA a‐IGZO TFT and the gold electrode. Three sections of the schematic are highlighted, and corresponding cross‐sectional images obtained using scanning electrode microscopy (SEM) and focused ion beam SEM (FIB‐SEM) are presented. Images include the gate stack of a SA a‐IGZO TFT (Figure 1e‐i); the edge of a recording gold electrode, sandwiched between the interlayer and top encapsulation SU‐8 layers (Figure 1e‐ii); and the lateral encapsulation protecting the edges of the µECoG array (Figure 1e‐iii). The last SU‐8 layer fully covers the sides of the thin‐film transistor stack, extending until the substrate, and preventing fluids from reaching sensitive layers of the stack, i.e., SiN_x_ and Al_2_O_3_ (Figure 1e‐iv).

The biocompatibility of the µECoG array was evaluated by assessing the cytotoxicity of each of its materials, as reported in the scientific literature (Table S1, Supporting Information). Regarding the semiconductor material, amorphous indium gallium zinc oxide, its biocompatibility has recently been reported in different mammalian cell types.^[^ ^30^ ^]^ In order to verify this, the cytotoxicity of a‐IGZO was evaluated following ISO 10 993‐5:2009 standard: *Biological evaluation of medical devices – Part 5: Tests for* in vitro *cytotoxicity*, where biological reactivity of a mammalian monolayer was quantified in response to extracts of a test sample containing a 24‐nm a‐IGZO layer. Cell cultures exposed to the extracts presented a cell viability of 96% after a 48‐hour incubation period (Table S2, Supporting Information). Based on the evaluation criteria, for which samples with a cell viability lower than 70% are considered cytotoxic, a‐IGZO was determined to be non‐cytotoxic.

The µECoG arrays can be operated in two different modes, depending on the way the select lines are switched. In addressing mode (or channel‐selection multiplexing), a subset of electrodes of interest is selected and data from only these electrodes is recorded for an extended period of time. Even though not all electrodes can be read simultaneously, operating in addressing mode allows to sequentially record from blocks of electrodes while still employing standard acquisition systems for electrophysiology. Additionally, recording in addressing mode imposes less constraints on the electrode and TFT dimensions, in terms of required impedance and noise values, allowing to reduce the pixel size. Arrays to be used in addressing mode followed a distributed design, with blocks of electrodes that could be addressed by the corresponding select line. Each block of electrodes was separated in space, which allowed to record from different brain areas of interest. As an example, we designed a µECoG array containing 62 recording electrodes, divided in two subarrays of 31 electrodes each and spaced 2 mm apart (Figure S2a, Supporting Information). Electrodes in each subarray could be addressed by activating one of the two select lines (multiplexing ratio 2:1). Recording pixels were formed by an a‐IGZO TFT with channel width (W) and length (L) of 50 µm and 3 µm, respectively, and a gold electrode 100 µm in diameter, with a 250‐µm pitch.

In time‐division multiplexing mode, all the electrodes sharing a single data line are sequentially sampled one after the other faster than the dynamics of the measured signal, enabling recording from all pixels virtually simultaneously. Arrays to be used in time‐division multiplexing mode follow a matrix configuration with 16×16 recording pixels, with an overall dimension of the recording area of 8×8 mm^2^ (Figure 1c). Recording pixels were formed by an a‐IGZO TFT with W/L  =  300 µm/3 µm, and a gold electrode 300 µm in diameter, with a 500‐µm pitch (Figure 1d). A smaller version of the multiplexed array containing 8×8 pixels was also fabricated, facilitating initial testing in small animal models (Figure S2b, Supporting Information).

### 2.2 In Vitro Characterization of Multiplexed µECoG Electrode Arrays

**Figure 2:**
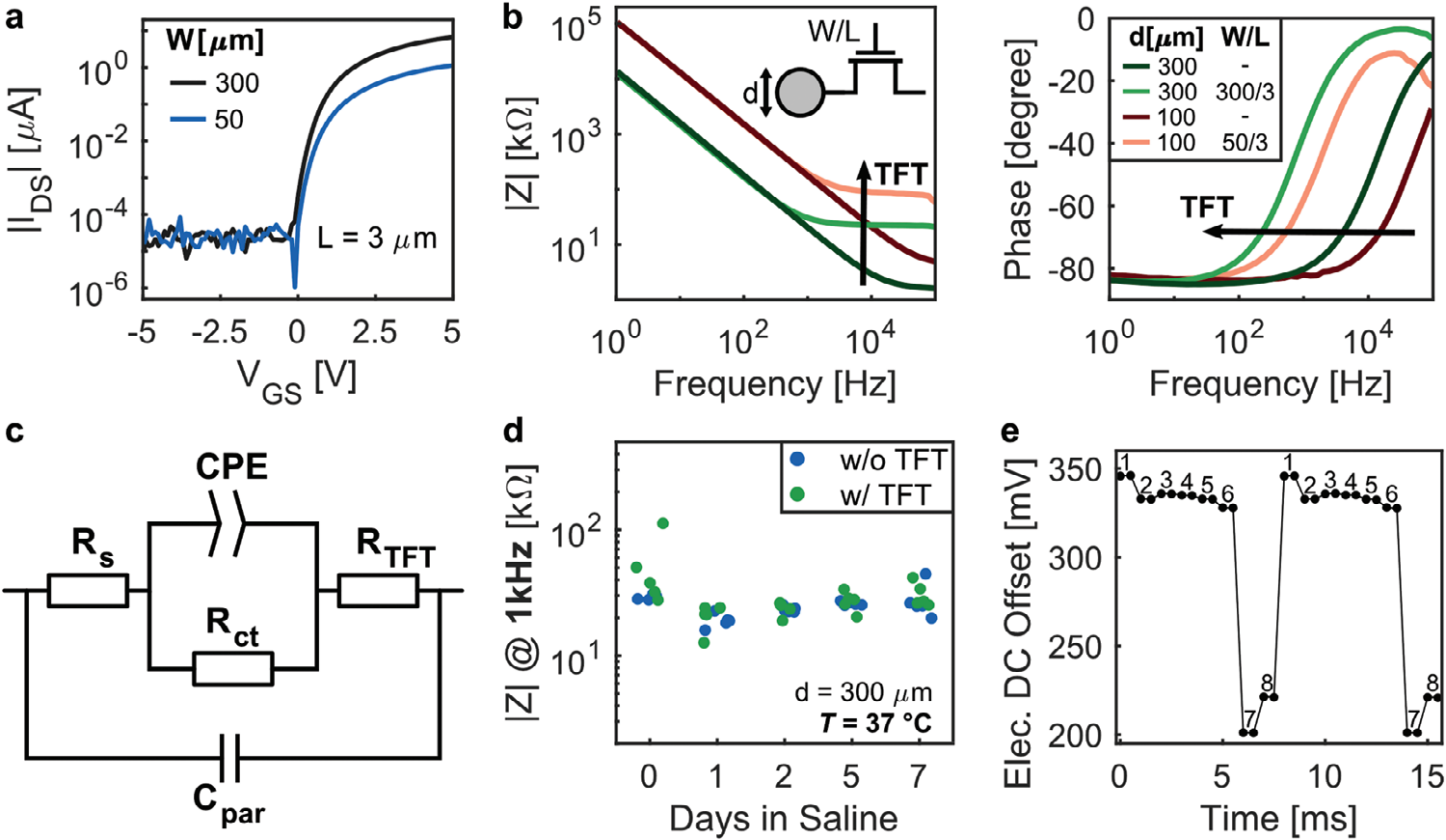
Electrical characterization of the µECoG array and of its principal components. a) Transfer characteristics (I_DS_ versus V_GS_) of free‐standing thin‐film transistors for two different channel widths (W/L  =  300 µm/3 µm, and W/L  =  50 µm/3 µm), at V_DS_ of 100 mV. b) Bode plot (magnitude and phase) of the electrochemical impedance of various pixel designs formed by a gold electrode with and without the presence of a select transistor for two different electrode diameters (300 µm and 100 µm). c) Equivalent electrical circuit model used for the interpretation of the impedance spectra. The model corresponds to a modified version of the Randles circuit with the addition of a resistors in series (*R_TFT_*), modeling the *R_ON_* of the TFT, and a capacitance in parallel (*C_par_*), accounting for parasitic capacitances. d) Lifetime assessment of a µECoG array kept in phosphate buffer saline at 37°C for up to 7 days. Electrode impedance at 1 kHz was monitored over time for electrodes with and without the presence of a switch TFT present in the same test array (300‐µm electrode diameter, W/L  =  300 µm/3 µm, *n*  =  5 per pixel type). e) Electrode DC offset of 8 multiplexed electrodes switched at 1 kHz. Numbers 1 to 8 represent the selected electrode.

The electrical and electrochemical characterization of the recording pixel was carried out for each of its components (the thin‐film transistor and the electrode) independently and in combination, and the impact of adding the TFT was evaluated.

a‐IGZO TFTs were characterized by measuring their transfer characteristics (I_DS_ – V_GS_ curves) for different transistor channel widths (**Figure** **2**a; Figure S3a, Supporting Information) and by calculating the corresponding ON resistance (*R_ON_*) at the operation bias of the active µECoG arrays (V_GS_  =  +5 V) (Figure S3b, Supporting Information). Mapped TFTs presented small spread in their transfer characteristics, both for TFTs with W/L  =  300 µm/3 µm (Figure S3c, Supporting Information: *R_ON_*  =  16.3 ± 3.1 kΩ, *V_ON_*  =  0.2 ± 0.3 V, *n*  =  67), and for TFTs with W/L  =  50 µm/3 µm (Figure S3d, Supporting Information: *R_ON_*  =  95.1 ± 12.5kΩ, *V_ON_*  =  0.5 ± 0.3 V, *n*  =  15). Reducing the transistor channel width from 300 to 50 µm caused a six‐fold decrease in the ON current and a corresponding increase in the ON resistance.

Recording pixels were characterized through electrochemical impedance spectroscopy and the impact of the TFT on the impedance was assessed. Impedance spectra of individual pixels with and without the presence of a TFT were measured for two combinations of electrode diameter and transistor dimensions (300‐µm electrode, transistor dimensions W/L  =  300 µm/3 µm; and 100‐µm electrode, transistor dimensions W/L  =  50 µm/3 µm) (Figure 2b). As expected, larger electrodes offered lower impedances. The addition of the TFT did not have an impact on the impedance magnitude (|Z|) at lower frequencies, where impedance was dominated by the double‐layer capacitance of the electrode‐electrolyte interface. However, at higher frequencies, the presence of the TFT caused an increase of the impedance magnitude, equivalent to the ON resistance of the transistor. A shift in impedance phase toward lower frequencies was also observed when the TFT was present, indicating an earlier transition from a capacitive to a resistive coupling regime. Impedance measurements were extended to pixels with varying transistor channel widths (W  =  30 µm to 3000 µm) for both 300‐µm (Figure S4a, Supporting Information) and 100‐µm (Figure S4b, Supporting Information) electrode diameters. Overall, a similar trend in the impedance spectrum was observed for both electrode sizes, with decreasing impedance values at higher frequencies as transistor channel width increases.

For the arrays operated in addressing mode, an electrode diameter of 100 µm was selected, along with a transistor dimension of W/L  =  50 µm/3 µm. For the 16×16 arrays operated in time‐division multiplexing mode, the chosen dimensions were of 300 µm for the electrode and a W/L  =  300 µm/3 µm for the transistor. Transistor dimensions were chosen as a tradeoff between the area footprint and the minimization of its ON resistance. Similarly, the electrode size was chosen as a compromise between electrode impedance and electrode noise on the one hand, and spatial resolution on the other. Pixel dimensions for the 16×16 arrays were chosen to be larger to compensate for the increase of the overall noise due to the time‐division multiplexing operation. The overall array dimensions were a compromise between maximizing the electrode count, device electrical performance, and the compatibility with small animal models targeted for validation (mice). All pixels of a 16 × 16 array were measured, and the impedance spectra showed low variation (|Z|_(1 kHz)_  =  24.5 ± 6.4 kΩ) across the entire array, with just two defective pixels (Figure S4c, Supporting Information).

We modeled the impedance spectra of the different recording pixels using an equivalent circuit based on the Randles circuit (Figure 2c). The standard Randles model includes a series resistance (*R_s_*), corresponding to the solution and track resistances; a constant phase element (*CPE*), accounting for the double‐layer capacitance; and a charge transfer resistance (*R_ct_*), modeling any faradaic reactions. In addition, the modified model included an extra resistor in series (*R_TFT_*), corresponding to the ON resistance of the TFT, as well as a capacitance in parallel (*C_par_*), accounting for the solution capacitance or parasitics, for which the effect was seen at high frequencies. Using the proposed model, the measured spectra were accurately fitted for both electrodes with and without a TFT, as exemplified by Bode plots, Nyquist plots and Nyquist plots of the complex capacitance (Figure S5 and Table S3, Supporting Information). The modeling results of the pixel impedance confirmed that the incorporation of the TFT simply translates into an increase of the series resistance in the Randles circuit, equivalent to the ON resistance of the TFT. This effect was captured with the additional resistive element *R_TFT_*.

The stability and reliability of the fabricated µECoG arrays were tested in vitro, where the arrays were kept in phosphate buffered saline (PBS, 1X) at physiological temperature (37°C). For this test, we employed a dedicated test array included electrodes with and without a switch TFT (300 µm in diameter, W/L  =  300 µm/3 µm), for which the electrode impedance was monitored over time (*n*  =  5 per electrode type for 1 test array). The impedance was stable over a period of 7 days, with no difference between the two electrode types (Figure 2d). Arrays failed due to liquid penetration through the connector pads and possibly through weak points in the SU 8 encapsulation. No signs of layer delamination or of liquid ingress through the sides of the array were observed. Lifetime assessment of the arrays demonstrated their capability to be functional for at least 7 days in vitro, paving the way for their use in short‐term chronic experiments. Further work is needed to validate the encapsulation strategy and its future translation to a chronic setting.

In acquisition systems where several electrodes are multiplexed into a single shared readout line, the multiplexed signal is seen as a step‐like waveform by the readout channel.^[^ ^31^ ^]^ This waveform is dominated by the large amplitude of the distinct Electrode DC Offsets (EDOs), which are superimposed to the measured neural activity. Characterized EDOs of 8 multiplexed electrodes switched at 1 kHz show the step‐like behavior (Figure 2e), with EDO values in the hundreds of millivolts for our 300‐µm gold electrodes (EDO  =  397 ± 115 mV, *n*  =  256). Advantageously, the magnitude of the EDO seen at the input of the recording electronics will be reduced by the voltage divider formed between the electrode DC impedance and the input impedance of the readout channel. A large DC electrode impedance (≈500 MΩ for our 300 µm Au electrodes, see Table S3, Supporting Information), much larger compared to the input impedance of the readout electronics, will result in a substantial attenuation of the electrode open‐circuit potentials.^[^ ^32^ ^]^

### 2.3 In Vitro Recordings with Multiplexed µECoG Electrode Arrays

#### 2.3.1 In Vitro Recordings in Addressing Mode of Operation

**Figure 3:**
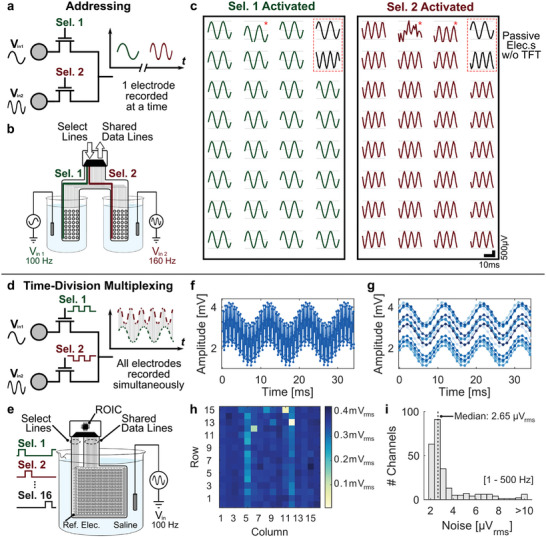
In vitro characterization of the recording capabilities of µECoG arrays in addressing and time‐division multiplexing modes of operation. a) Schematic diagram illustrating the operation of the µECoG arrays in addressing mode, where pixels sharing the same data line can be addressed by the corresponding select line (i.e., Sel.1 and Sel.2). b) Diagram of the characterization setup in saline for a µECoG array in addressing mode and c) corresponding in vitro recordings. A probe formed by two subarrays of electrodes was employed, in which data lines were shared between pairwise electrodes located in each of the subarrays. Each subarray incorporated 30 multiplexed electrodes (100 µm in diameter, transistor dimensions of W/L  =  50 µm/3 µm), along with an additional electrode without a TFT (100 µm in diameter). The subarrays were placed in separate solutions, receiving distinct sinusoidal input signals (V_in 1_  =  1 mV_pp_, 100 Hz, and V_in 2_  =  1 mV_pp_, 160 Hz). Activating one of the select lines enabled recording from all 30 multiplexed electrodes from the respective subarray. Additionally, the two electrodes without TFTs (red dashed rectangle), each located in a different subarray, continuously recorded from the corresponding solutions, independently of the activated select line. Three defective pixels are marked with a red star. d) Schematic diagram illustrating the operation of the µECoG arrays in time‐division multiplexing mode. Pixels sharing the same data line are switched rapidly by applying pulse trains to the select lines (i.e., Sel.1 to Sel.16), allowing to record signals from all multiplexed electrodes virtually simultaneously. e) Diagram of the characterization setup in saline for a µECoG array in combination with the ROIC operating in time‐division multiplexing mode. A 256‐channel µECoG array was employed (16 × 16 pixels, 300‐µm electrode diameter, transistor dimensions W/L  =  300 µm/3 µm). All electrodes were placed in the same saline solution (V_in_  =  1 mV_pp_, 100 Hz) and signals from the multiplexed data lines were recorded using the dedicated ROIC. f) Output from one super‐channel recording 16 multiplexed pixels sharing a single data line, and g) corresponding demultiplexed signals. h) RMS values measured for the full array for a 1 mV_pp_ (0.35 mV_rms_) input signal. i) Histogram of the input‐referred noise of the entire system (µECoG array + ROIC) measured in saline with the solution grounded.

We first validated the arrays operating in addressing mode, in which signals are recorded only from a subset of selected pixels (**Figure** **3**a). To easily test the capabilities of our arrays to record distinct signals from multiple electrodes sharing a single data line, we employed a probe formed by two subarrays of electrodes, separated in space (Figure S2a, Supporting Information). The probe contained pairs of addressable electrodes located in each subarray, offering a multiplexing ratio of 2:1. Each subarray contained 30 addressable electrodes in an 8 × 4 matrix configuration. An electrode not connected to a TFT, was also included in each subarray to serve as comparison with the addressable pixels. By activating one of the select lines (V_sel ON_  =  +5 V, V_sel OFF_  =  −2 V), we could record from the corresponding subarray employing a commercial electrophysiology acquisition system (RHD2132 recording headstage, Intan Technologies).

Recordings were done by placing each subarray in separate saline solutions, allowing each of them to receive distinct input signals (Figure 3b). We then applied two sinusoidal signals with different frequency (V_in 1_  =  1 mV_pp_, 100 Hz, and V_in 2_  =  1 mV_pp_, 160 Hz) to the two solutions in order to demonstrate that the different subarrays could be addressed, recording signals only from selected electrodes (Figure 3c). The recorded signals changed between a sinusoidal at 100 Hz (subarray on the left) or one at 160 Hz (subarray on the right), depending on the select line activated. Data from each of the subarrays was recorded sequentially in time, with the recording being paused when switching between electrodes. The recordings were restarted after a brief pause of a couple of seconds, which was necessary for the system to stabilize. The channels connected to the electrodes without a TFT, located on the top right corner of the subarrays, always recorded from the same solution, independently of the activated select line. These electrodes served as controls, recording from the solution in which the corresponding subarray was placed. The subarray on the left had one defective pixel, and the one on the right had two.

Measurements in the time and frequency domains showed no discernible differences, whether recorded using electrodes with or without a switch TFT (Figure S6, Supporting Information). Crosstalk between subarrays was measured to be ‐46 dB for electrodes without a TFT and ‐53 ± 2 dB (*n*  =  29) for addressed electrodes sharing a data line, showing no noticeable difference between both scenarios. Noise values, calculated from the integrated power spectral density (1 – 500 Hz), were of 2.8 ± 0.2 µV_rms_ (*n*  =  2) for the electrodes without a TFT and 3.5 ± 0.9 µV_rms_ (*n*  =  57, after removing three defective pixels) for the addressed electrodes with the TFTs. Both measurements include the noise contribution of the recording board, 2.5 ± 0.2 µV_rms_ (*n*  =  32). The individual contribution of the 100‐µm gold electrode and the 50 µm/ 3 µm a‐IGZO TFT to the overall noise was calculated to be 1.3 µV_rms_ and 2.1 µV_rms_, respectively. In conclusion, our active µECoG arrays can sequentially record from multiple electrodes sharing a single data line with similar performance as standard electrodes without a TFT, thanks to the incorporation of a select TFT in each recording pixel.

#### 2.3.2 In Vitro Recordings in Time‐Division Multiplexing Mode of Operation

The µECoG arrays can also be operated in time‐division multiplexing mode, where pixels sharing the same data line are sampled sequentially by switching between the select lines at a frequency of 1 kHz, allowing to record signals from all electrodes virtually simultaneously (Figure 3d). Time‐division multiplexing directly at the electrode array presents additional challenges to the readout electronics compared to non‐multiplexed systems, mainly the switched EDOs and the aliasing of out‐of‐band noise.^[^ ^26^ ^]^ The acquisition electronics must record µV‐level signals in the presence of large and unwanted electrode DC offsets, which cannot be filtered out as in standard AC‐coupled systems and can easily saturate the readout electronics. Additionally, in multiplexed recording systems, the acquisition bandwidth must be at least *N* times larger than the signal band (with *N* being the multiplexing ratio), which leads to an increase in the noise bandwidth. In the reconstruction process, demultiplexing the recorded signal by subsampling it at a frequency (*f_ch_*) leads to noise components above the signal band (*f_ch_*/2) to fold back into the band of interest. This results in at least an *N*‐fold increase in the total in‐band noise power in the final demultiplexed output (assuming a white noise spectrum and a noise bandwidth of *N ∙ f_ch_*/2). See supporting information for an extended explanation, Figure S7 (Supporting Information). Advantageously for µECoG recordings, the magnitude of the folded noise can be kept low in comparison to penetrating probes recording action potentials, due to the larger surface electrodes offering lower noise as well as the narrower signal bandwidth (1 – 500 Hz).

To tackle these challenges inherent of time‐division multiplexed recordings, we previously developed a dedicated readout integrated circuit in a 22‐nm fully depleted silicon on insulator (FDSOI) CMOS technology.^[^ ^25^, ^26^ ^]^ The ROIC is a low‐noise, wide‐bandwidth, and area‐power‐efficient IC capable of handling multiplexed signals. Our designed ROIC implements a mixed‐signal feedback EDO compensation loop that can cancel the modulated EDOs (up to 160 mV_pp_), avoiding the saturation of the readout electronics in the presence of large EDOs. Additionally, the readout channel follows a close‐loop incremental‐ΔΣ ADC architecture that acts as an inherent anti‐aliasing filter, thanks to its windowed integration. This minimizes the noise folding by limiting the noise bandwidth to a minimum of *N*‐times the signal bandwidth. Defining a signal bandwidth between 1 Hz and 500 Hz, a sampling frequency of 1 kHz, and having a multiplexing ratio of 16, the ADC filters the noise for frequencies above 8 kHz. However, noise power between 500 Hz and 8 kHz is not filtered out and will be folded into the signal band when demultiplexing. To mitigate this excess noise, the thermal noise floor of both the recording electronics as well as the electrode array must be carefully considered and minimized in the design. Hence, the electrode impedance as well as the switch ON‐resistance was kept as low as possible by sizing the electrodes and TFTs accordingly (300‐µm electrode, and TFT dimensions W/L  =  300 µm/3 µm).

The ROIC was designed to interface with a 16 × 16 active µECoG array (Figure 1c) implementing a 16:1 multiplexing ratio, which allows to record from all the 256 electrodes using only 16 multiplexed channels (termed as super‐channels), and 16 digital select lines. The high‐level architecture of the complete proposed system, formed by the µECoG array and the dedicated ROIC can be found in Figure S8a (Supporting Information). Each super‐channel is responsible for the signal acquisition and digitalization, as well as the EDO compensation, of 16 multiplexed electrodes (the block diagram of the super‐channel is presented in Figure S8b, Supporting Information). The ROIC integrates all the required analog signal processing, biasing, and digital control circuits for the super‐channels, as well as the digital logic to control the TFT switch matrix. However, the power‐management unit driving the TFTs was implemented off‐chip, since it requires higher operating voltage levels of −2 and +5 V. Thanks to the sharing of the readout super‐channel by multiple electrodes, the ROIC presents a very low effective silicon area and power per electrode channel of 0.001 mm^2^ and 1.61 µW, respectively.

We validated the complete system (the µECoG array along with the ROIC) in vitro by recording in time‐division multiplexing mode (Figure 3e) from a saline solution biased with a test sinusoidal signal (V_in_  =  1 mV_pp_, 100 Hz; select lines: V_sel ON_  =  +5 V, V_sel OFF_  =  ‐2 V, switching frequency  =  1 kHz, duty cycle  =  1/16). Each super‐channel acquired multiplexed signals from 16 pixels sharing a single data line (Figure 3f), which when demultiplexed allowed the recovery of the original sinusoidal input signal (Figure 3g). The different offsets observed in the demultiplexed signals correspond to the residual EDOs after compensation, which are low enough to avoid the saturation of the acquisition system. Evaluating the performance of the full array, Figure 3h presents a color map of the root mean square (RMS) values of the recorded signals, measured for a 1 mV_pp_ (0.35 mV_rms_) input signal. Some channels presented a lower amplitude, attributed to defective pixels (electrode or TFT) in the µECoG array or to issues in the recording super‐channel. The noise of the complete system operating in time‐division multiplexed mode was measured in saline with the solution grounded (Figure 3i), with the integrated input‐referred noise yielding a median noise level of 2.65 µV_rms_ and an average value of 3.31 ± 1.62 µV_rms_ (1 – 500 Hz, n  =  250, after removal of 6 defective pixels with noise > 10 µV_rms_). The ROIC alone presented an in‐band integrated noise of 1.55 ± 0.2 µV_rms_ (1 – 500 Hz, *n*  =  256). Consequently, the contribution of the active µECoG array, including the noise folding, was only ≈2.9 µV_rms_ in average. The lower noise, compared to existing active µECoG arrays,^[^ ^19^, ^20^, ^21^, ^22^, ^33^ ^]^ is achieved by the simple pixel implementation, with only the switch TFT on the flexible array, limiting the magnitude of the flicker noise contributions from the flexible transistors.

These experiments demonstrate the capability of our active µECoG array to acquire signals in time‐division multiplexing mode. The dedicated ROIC tackles the specific challenges originating from multiplexed recordings and enables the acquisition of hundreds of electrodes without increasing proportionally the number of recording lines.

### 2.4 In Vivo Validation

We successfully validated our multiplexed µECoG arrays in vivo through acute electrophysiological recordings in anesthetized mice, monitoring both spontaneous neural activity and evoked responses elicited by peripheral electrical stimulation of the animal's paws.

#### 2.4.1 In Vivo Recordings in Addressing Mode

**Figure 4:**
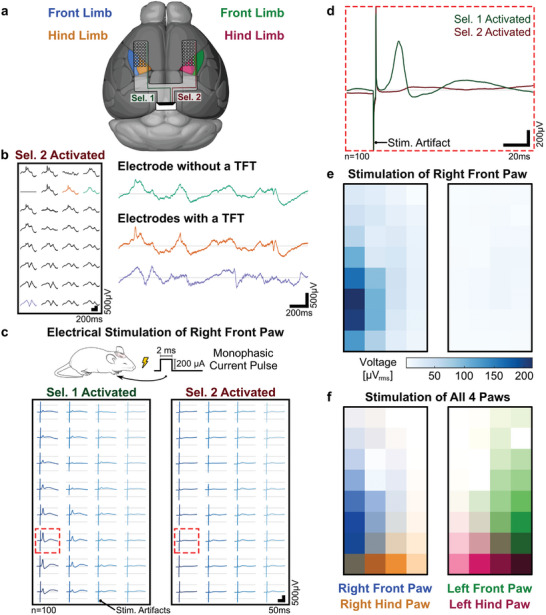
In vivo validation of the µECoG arrays in addressing mode. a) Representation of the placement over the cortical surface of a µECoG array formed by two subarrays (8 × 4 pixels per subarray, 100‐µm electrode diameter, 250‐µm pitch). Each of the subarrays was placed in a different hemisphere, partially covering the somatosensory regions representing the front and hind limbs. b) Recorded spontaneous activity from all the addressable electrodes when selecting one of the subarrays, and magnification of three of those electrodes: one electrode without a TFT (green), an adjacent electrode incorporating a switch TFT (orange) and a distant electrode with a TFT (purple). c) Averaged response of the somatosensory evoked potentials elicited by the electrical stimulation of the right front paw of an anesthetized mouse (monophasic current pulses: 200 µA in amplitude, 2 ms in duration, 1 Hz frequency, *n*  =  100). Each subarray was recorded sequentially. The subarray activated with select line #1 was located in the hemisphere contralateral to the stimulated paw, while the subarray activated with select line #2 was in the ipsilateral hemisphere. The electrode with the strongest response, and its corresponding pair in the other subarray are highlighted (red dashed square). d) Average response of the highlighted electrodes: two addressable electrodes located in different subarrays but sharing a single data line and readout channel. e) Localized gamma response to stimulation of the right front paw. The color map indicates the RMS instantaneous power of the averaged responses filtered between 30 Hz and 500 Hz in an interval 10 to 50 ms after the stimulation onset. f) Overlay of the normalized gamma responses to the sequential stimulation of all four paws. Color intensities indicate the magnitude of the RMS instantaneous power measured for each stimulated paw (right front paw = blue, left front paw = green, right hind paw = orange, and left hind paw = fuchsia).

A µECoG array formed by two subarrays was implanted epidurally over the cortical surface. Each of the subarrays was placed in a different hemisphere, partially covering the somatosensory areas representing the front‐ and hind limbs (**Figure** **4**a). Spontaneous activity was recorded from either hemisphere by selecting the corresponding subarray (Figure 4b). The array captured localized neural activity, with electrodes 250 µm apart presenting distinct traces. Nearby electrodes presented higher correlation, but as the distance between electrodes increased, the shape of the recorded waveform clearly diverged. No noticeable difference was observed between the control electrode without a TFT and neighboring electrodes incorporating the TFTs.

To record controlled and localized physiological signals, distinguishable between both subarrays, we elicited somatosensory evoked potentials (SSEPs) by peripheral electrical stimulation. The front paw of a mouse was electrically stimulated using monophasic current pulses (200 µA in amplitude, 2 ms in duration, 1 Hz frequency, *n*  =  100) (Figure S9a, Supporting Information). A strong evoked response was observed when measuring from the subarray located in the contralateral hemisphere, even for single trials (Figure S9b,c, Supporting Information). The amplitude and timing of the evoked response differed between successive stimulation trials (Figure S9d, Supporting Information), but the average signal (*n*  =  100) exhibited a well‐defined response (Figure S9e–g, Supporting Information). By recording sequentially from each of the two subarrays, the evoked response in both hemispheres could be compared (Figure 4c). Recorded evoked potentials showed the strongest response in the subarray contralateral to the stimulated paw, with an amplitude of the first peak of ≈580 µV, occurring ≈20 ms after stimulation onset. Responses in the ipsilateral hemisphere were much weaker, slower, and broader, only noticeable after averaging multiple trials, with an amplitude of the first peak of ≈85 µV, occurring ≈30 ms after the stimulation onset. Thanks to the channel selection multiplexing, it was possible to record sequentially different signals from two addressable electrodes located in different subarrays but sharing a single data line and readout channel without crosstalk between them (Figure 4d).

Spatial localization of the evoked responses can be better represented looking at the broadband gamma activity, as the high‐frequency components of the evoked response are more localized than the lower‐frequency activity.^[^ ^13^ ^]^ Color maps reflecting the spatial distribution of the measured signals were generated by bandpass filtering the measured responses in the frequency range between 30 Hz and 500 Hz, and calculating its RMS instantaneous power after stimulation (Figure S10, Supporting Information). The spatial distribution of the recorded evoked potentials shows that the response was highly localized to the contralateral hemisphere, and its power decreased sharply when moving away from the focal point (Figure 4e). Overall, the response recorded by each subarray exhibited different patterns, confirming the ability of our active µECoG arrays to capture diverse biological signals from selected addressable electrodes that incorporate the a‐IGZO TFTs.

By extending the stimulation to all four paws, distinct and robust SSEPs could be observed for each of the paws when stimulated sequentially (Figure S11, Supporting Information). Recording from both subarrays enabled monitoring the responses in both hemispheres. The amplitude of the SSEPs varied depending on the stimulated paw, as well as their localization, which reflected the underlying brain organization, with strong and localized responses in the hemisphere opposite to the stimulated paw (Figure S12, Supporting Information). Overlaying the responses for each stimulation condition generates a comprehensive color map that visually represents the spatial distribution of the distinct evoked responses resulting from the stimulation of all four paws (Figure 4f).

#### 2.4.2 In Vivo Recordings in Time‐Division Multiplexing Mode

A 16 × 16 actively multiplexed µECoG array was implanted epidurally over the cortical surface, covering a large fraction of both hemispheres (≈1 × 1 cm^2^). Photographs depicting the experimental setup used for the in vivo recordings are presented in Figure S13 (Supporting Information).

##### Spontaneous Activity

**Figure 5:**
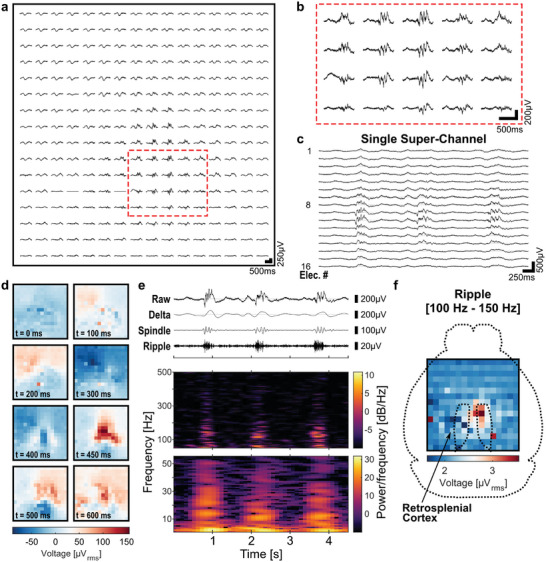
In vivo recordings of spontaneous activity using a 16 × 16 µECoG array in time‐division multiplexing mode. a) Recorded spontaneous activity showing the spatial distribution of measured physiological oscillations under anesthesia for all the 256 electrodes of the multiplexed array, and b) for a subset of electrodes capturing localized events. c) Spontaneous activity captured by 16 multiplexed electrodes sharing a single recording super‐channel. d) Spatiotemporal dynamics of spontaneous activity (650 ms timespan with 50 ms epochs). For each frame, the pixels’ color indicates the mean voltage calculated in the corresponding time bin. e) Raw and filtered time traces for a selected electrode capturing physiological oscillations. Signal filtered in the delta (1 Hz – 4 Hz), spindle (10 Hz – 16 Hz), and ripple (100 Hz – 150 Hz) bands. Spectrograms of presented time trace in the frequency ranges 1 to 55 Hz and 50 to 500 Hz. f) RMS instantaneous power of spontaneous activity in the ripple band, reflecting localized ripple activity over midline cortical structures (129 seconds of recording).

Spontaneous electrophysiological activity was recorded from the cortical surface (**Figure** **5**a), with localized physiological events measured by electrodes spaced 500 µm from each other (Figure 5b). Recording super‐channels in the developed ROIC captured neural activity from groups of 16 multiplexed electrodes sharing a single data line, with each demultiplexed trace presenting distinct signals (Figure 5c). Interestingly, some electrodes present bursts of transient high‐frequency oscillations (HFOs), similar to reported signals captured by high‐resolution µECoG arrays in rodents.^[^ ^34^ ^]^ The recorded HFOs could have been related to hippocampal‐cortical coupling involved in memory transferring and retrieval,^[^ ^34^, ^35^ ^]^ or could have simply been induced by the employed anesthetics (ketamine/medetomidine), as ketamine has been reported to increase the power of these high‐frequency oscillations in different brain areas.^[^ ^36^, ^37^ ^]^ Spatiotemporal dynamics of the recorded activity can be better visualized by looking at sequential frames representing the average voltage over a given epoch (Figure 5d, 650 ms timespan with 50 ms epochs). The large‐scale coverage offered by the arrays allowed capturing complex dynamics occurring locally and globally over the cortical surface.

Exploring the recorded oscillations, traces were filtered in the delta (1 Hz–4 Hz), spindle (10 Hz–16 Hz), and ripple (100 Hz–150 Hz) frequency bands for a selected electrode (Figure 5e) and a subset of electrodes (Figure S14a–d, Supporting Information). Filtered signals showed clear oscillations localized in time, frequency, and space. Ripple activity was coupled to slower, large‐amplitude local field potentials (LFPs), and ripple events tended to co‐occur along cortical spindles at the transition from throughs to crests of slower delta waves, as previously reported.^[^ ^34^, ^38^ ^]^ The spectrogram of a selected electrode revealed wide spectral content of the recorded oscillations, extending from the lower frequencies up to the ripple band. In the same way, when comparing the power spectral densities of electrodes with and without high‐frequency oscillations, electrodes capturing the oscillations showed a considerable increase in power across a wide frequency range (Figure S14e, Supporting Information). Specifically, the power spectrum was higher between 5 to 40 Hz and between 90 to 150 Hz, with peak frequencies at 13 Hz and 120 Hz, respectively.

Looking at the spatial distribution of the different oscillations, delta waves extended over most of the array, while spindle and ripple activity were localized to midline cortical structures, as quantified from the RMS instantaneous power of the filtered signal over the corresponding frequency bands (Figure S15a–c, Supporting Information). Specifically, electrodes that presented activity in the ripple band were centered around midline structures and the right retrosplenial cortex, covering an area of ≈ 2  ×  1.5 mm^2^ of cortical surface (Figure 5f). These observations are in line with previous work, where cortical oscillations with peak frequencies ranging from 100 to 150 Hz have been reported in different cortical areas,^[^ ^38^ ^]^ with prevalence in associational areas, such as the posterior parietal cortex and midline structures, such as cingulate and retrosplenial cortices.^[^ ^34^ ^]^ Focusing on the physiological features of observed cortical ripples, they presented a median duration of 96 ± 58 ms (Figure S15d, Supporting Information), a mean peak frequency of 134 ± 9 Hz (Figure S15e, Supporting Information), and an occurrence of 0.75 events per second (*n*  =  97 ripples from 129 s of recording in 1 mouse).

##### Somatosensory Evoked Potentials

**Figure 6:**
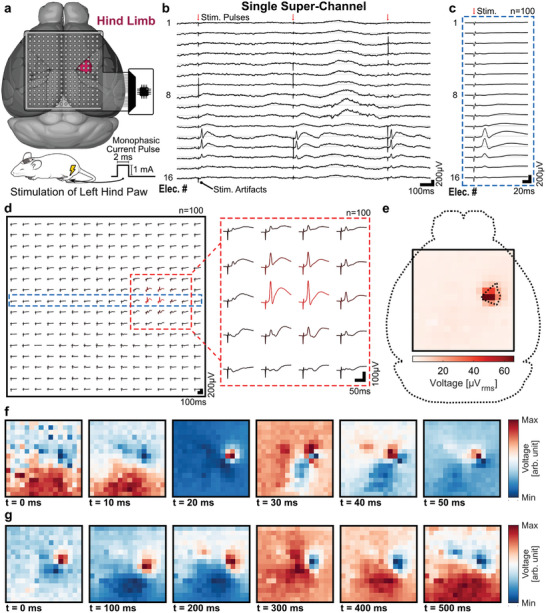
In vivo recordings of somatosensory evoked potentials using a µECoG array in time‐division multiplexing mode. a) Representation of the placement of a 16 × 16 actively multiplexed µECoG array over the cortical surface of an anesthetized mouse, highlighting the somatosensory region representing the hind limb in the right hemisphere. The left hind paw of the mouse was electrically stimulated using monophasic current pulses (1 mA in amplitude, 2 ms in duration, and with a frequency of 1.5 Hz) to elicit an evoked response in the contralateral somatosensory cortex. b) Evoked responses captured by 16 multiplexed electrodes sharing a single recording super‐channel, for three stimulation trials. c) Averaged evoked response for a selected super‐channel, and for d) all 256 electrodes of the multiplexed array (*n*  =  100). In the grid representation, the selected super‐channel presented in c) is highlighted (blue dashed rectangle). A magnification of the electrodes around the elicited response is included. e) Localized gamma response to stimulation of the left hind paw. The color map indicates the RMS instantaneous power of the averaged responses filtered between 30 Hz and 500 Hz in an interval 10 to 50 ms after the stimulation onset. f) Spatiotemporal dynamics of the averaged response for the first tens of milliseconds (55 ms timespan with 5 ms epochs) and g) the first hundreds of milliseconds (550 ms timespan with 50 ms epochs) after electrical stimulation of the left hind paw. For each frame, the pixels’ color indicates the mean voltage calculated in the corresponding time bin. The range of the color bar is not shared between frames.

We recorded somatosensory evoked potentials using the actively multiplexed µECoG array. The left hind paw of a mouse was electrically stimulated using monophasic current pulses (1 mA in amplitude, 2 ms in duration, 1.5 Hz frequency, *n*  =  100) to elicit an evoked response (**Figure** **6**a). Multiplexed electrodes, sharing a data line and a single recording super‐channel, measured distinct signals without any sign of crosstalk and properly captured the SSEPs after the electrical stimulation (Figure 6b). The averaged response of a selected super‐channel showed a clear evoked response, only measured by a few of the multiplexed electrodes (Figure 6c). The SSEP was clearly elicited in single trials (Figure S16a,b, Supporting Information), with minor differences in the measured SSEPs between trials (Figure S16c,d, Supporting Information). Recordings from the whole array showed that the SSEP presented the strongest response over a set of electrodes located in the hemisphere contralateral to the stimulated paw (Figure 6d). The first peak of the contralateral evoked potential had an amplitude of ≈190 µV, occurring ≈20 ms after the stimulation onset, while the ipsilateral hemisphere had a weaker response of just ≈10 µV, occurring ≈35 ms after the stimulation. The lower amplitudes of the measured SSEPs compared to the ones measured with the addressable array in the previous section could be explained by multiple factors, including: the spatial average caused by the larger recording electrodes used in the 16 × 16 array (300 µm vs 100 µm), the possibility of not measuring from the exact location of the brain due to the coarser pitch of the array (500 µm vs 250 µm), or simply by differences between experiments, like the placement of the needle electrodes used for stimulation or the depth of the anesthesia at the moment of the recording. The spatial distribution of the evoked responses can be better visualized with a color map of the RMS instantaneous power of the signal's broadband gamma activity (Figure S17, Supporting Information), which shows a highly localized response spanning only 2 to 3 electrodes (1 mm to 1.5 mm coverage) and centered around the contralateral front limb region (Figure 6e).

Finally, the spatiotemporal dynamics of the elicited SSEP can be studied by looking at sequential frames representing the mean voltage at given epochs after the electrical stimulation (Figure 6f, 55 ms timespan with 5 ms epochs). After stimulation of the left paw, a clear localized increase in voltage occurs on the contralateral hemisphere over the somatosensory cortex, followed 10 ms later by a fainter response in the same region of the ipsilateral hemisphere and a decrease of voltage over midline cortical structures. Not sharing the range of the color bar between frames allows to better visualize small variations in the signal; however, sharing the range can be useful for better comparison between epochs (Figure S18, Supporting Information). Expanding the analysis over a longer timespan after stimulation (600 ms timespan with 50 ms epochs) reveals interesting dynamics over the areas previously mentioned (Figure 6g; Figure S19, Supporting Information). For example, the initial positive peak observed in the contralateral hind limb region is followed by a broader negative response.

Overall, we have successfully recorded evoked potentials from the somatosensory cortex in response to peripheral electrical stimulation. Signal amplitude and power increased over baseline in specific electrodes in response to the stimulation of the hindpaw, reflecting clear differences in the recorded signal between multiplexed electrodes. These results demonstrate the capabilities of the arrays to record in a multiplexed fashion. With this, our multiplexed arrays present themselves as a powerful tool to study events occurring over the cortical surface with high spatiotemporal resolution and large spatial coverage.

### 2.5 Comparison to State‐of‐the‐Art Active µECoG Arrays

Compared to state‐of‐the‐art active µECoG arrays, our system exhibits competitive features, including lower noise levels across a broader frequency band, and the use of a well‐established flexible electronics technology based on metal oxide TFTs, with potential for large‐scale manufacturing (**Table** **1**). The co‐design of the µECoG alongside the dedicated ROIC allowed optimizing the system for a broad recording bandwidth while still maintaining favorable noise levels. By using a simple passive‐pixel architecture with a single select transistor at each recording site, we minimize the transistor's impact, resulting in lower noise levels compared to existing active µECoG arrays. In a similar approach, Seo et al. recently introduced an active µECoG array with a passive‐pixel architecture using silicon nanomembrane transistors, achieving competitive noise levels.^[^ ^33^ ^]^ The achieved noise values give us margin for increasing the multiplexing ratio in future array iterations, thereby expanding electrode count without incurring a substantial performance degradation in terms of noise. However, this increase will be limited by targeted noise levels, a decrease in input impedance of the ROIC caused by parasitic capacitances of larger arrays, and elevated crosstalk levels due to higher switching speeds.

**Table 1:** Overview of Proposed Thin‐Film, Multiplexed µECoG Array and Comparison to State‐of‐the‐Art.

Reference	Viventi 2011^[^ ^19^ ^]^	Chiang 2020^[^ ^20^ ^]^	Garcia 2020^[^ ^39^ ^]^	Cisneros 2021^[^ ^22^ ^]^	Seo 2023^[^ ^33^ ^]^	This Work
**Technology**	Si Nanomem.	Si Nanomem.	Graphene FET	Graphene FET	Si Nanomem.	a‐IGZO TFT
**Transducer**	Pt Electrode	Capacitively‐Coupled Elec.	Graphene FET	Graphene FET	Au Electrode	Au Electrode
**Pixel Architecture**	Active Pixel	Active Pixel	Single sensing FET	Single sensing FET	Passive‐Pixel	Passive‐Pixel
**Channel Count**	360	1008	32	1024	256	256
**Transducer Size**	300 × 300 µm^2^	100 × 180 µm^2^	50 × 50 µm^2^	50 × 50 µm^2^	34 × 7 µm^2^	300 µm in ⌀
**Pitch**	500 µm	250 µm	400 µm	400 µm	40 µm	500 µm
**Substrate Material**	Polyimide	Polyimide	Polyimide	Polyimide	Polyimide	Polyimide
**Thickness**	25 µm	29 µm	14 µm	8 µm	2 µm	20 µm
**Coverage**	10 × 9 mm^2^	9.24 × 9.2 mm^2^	4.8 × 1.6 mm^2^	1.3 × 1.3 cm^2^	2.3 × 0.3 mm^2^	8 × 8 mm^2^
**Multiplexing type**	Time Mux.	Time Mux.	Freq. Mux.	Time Mux.	Time Mux.	Time Mux.
**Multiplexing ratio**	18:1	36:1	4:1	32:1	8:1	16:1
**Noise** ^a)^ **Noise Bandwidth**	30 µV_rms_ (1–139 Hz)	58 µV_rms_ (2–100 Hz)	6.29 µV_rms_ (1–10 Hz)	32 µV_rms_ (1–100 Hz)	≈6 µV_rms_ ^c)^ (2–4.5k Hz)	2.65 µV_rms_ (1– 500 Hz)
**Sampling Rate per channel**	277 S s^−1^	434 S s^−1^	5 kHz Bandwidth	13.2 kS s^−1^	390 S s^−1^	1 kS s^−1^
**In Vivo Demonstration**	Acutely in Cats	Chronically in Rats (435 days), Acutely in Monkeys	Acutely in Rats	None, Only In Vitro^b)^	Acutely in Rats	Acutely in Mice

^a)^ Including contribution from recording electronics;

^b)^ A smaller version of these arrays with 64‐channels has been demonstrated acutely in rats;^[^ ^21^ ^]^

^c)^ Current sensing recording. Noise = 32.3 pArms, equivalent to ≈6 µVrms.

## 3 Conclusion

Here, we present a proof‐of‐concept for overcoming the wiring bottleneck faced by high‐density µECoG arrays through the integration of thin‐film electronics into flexible electrode grids. By incorporating a‐IGZO thin‐film transistors on each recording pixel, the number of recording electrodes can be substantially increased without linearly scaling the number of required traces or recording channels. Combining the array with our dedicated silicon readout integrated circuit, which tackles the specific challenges of time‐division multiplexed recordings, we demonstrated in vivo the capability of measuring from up to 256 electrodes virtually simultaneously employing only 16 readout channels. The complete system presents excellent electrical performance, with noise levels lower than existing active µECoG arrays. Furthermore, our approach is based on a mature thin‐film semiconductor technology, compatible with large‐scale manufacturing of highly uniform arrays, a prerequisite for technology dissemination. Our multiplexed µECoG array presents itself as a novel neuroscience tool capable of mapping large areas of the cerebral cortex with low noise and high spatiotemporal resolution. Validating the devices in larger brains (such as in non‐human primates or intraoperative human subjects) should be done next, as it would leverage the large‐area coverage offered by the technology. Future work focused on incorporating better performing electrode materials and enhancing the encapsulation could yield devices with even higher electrode count, larger area coverage and increased recording resolution suitable for long‐term chronic recordings.

## 4 Experimental Section

### Fabrication of µECoG Electrode Arrays

Fabrication started with the deposition of a 15‐µm‐thick polyimide layer (PI‐2611, HD MicroSystems) through spin‐coating on a 6‐inch glass carrier wafer, and subsequent curing at 350°C under nitrogen. An Al_2_O_3_ buffer layer (100 nm) was deposited by low‐temperature atomic layer deposition (ALD) over the polyimide foil substrate. As the active layer, 24 nm of a‐InGaZnO was deposited by Direct Current (DC)‐sputtering in 10% O_2_ in Ar and patterned by wet etch using oxalic acid to define the transistor active area. As gate dielectric, SiO_2_ (100 nm) was deposited at 250°C by plasma‐enhanced chemical vapor deposition (PECVD), followed by the sputtering of Mo (100 nm) as gate metal. The gate stack, formed by the metal and gate dielectric, was patterned using reactive ion etching with a SF_6_ and O_2_ plasma to pattern the Mo, and an Ar and CF_4_ plasma to pattern the SiO_2_. Subsequently, a SiN_x_ intermetal dielectric (200 nm) was deposited at 150°C by PECVD (PlasmaPro 100 ICPCVD, Oxford Instruments), followed by the opening of vias with dry etch using a SF_6_ and Ar plasma. A metal stack of sputtered Ti/Al/Ti (10 nm/50 nm/10 nm) (Nimbus 310, NEXX Systems) was patterned by lift‐off (Microstrip 2001, Fujifilm, for 1 hour at 80°C) and used as source/drain contacts. With this, the thin‐film transistors backplane was completed.

The process continued with the deposition of a 1.7‐µm‐thick interlayer dielectric made of the negative photoresist SU‐8 (SU‐8 2002, MicroChem) and subsequent ultraviolet (UV) patterning for the definition of vias and a trench that followed the contour of the arrays. Gold (100 nm) was sputtered (Lab 18 Thin Film Deposition System, Kurt J. Lesker Co.) and patterned through wet etching (Au Etchant TFA, Transene; diluted 10:1 in H_2_O, 260 s), forming the recording electrodes. The SiN_x_ intermetal dielectric and the Al_2_O_3_ buffer layer were etched away from the trench around the arrays by reactive ion etching using a SF_6_ and Ar plasma, and BCl_3_ and Cl_2_ plasma, respectively, while employing the previously deposited SU‐8 layer as a hard mask. As the final layer, a second SU‐8 layer, 1.2‐µm thick, was deposited after an oxygen plasma activation step (100 W, 5 min), used to increase layer adhesion. The SU‐8 was patterned by photolithography, with openings over the metal electrodes, contact pads, and probe contour, and formed the top encapsulation of the arrays. A curing step at 130°C for 10 minutes was performed to increase the adhesion between the two SU‐8 layers.

After the stack was finalized, fabricated wafers were mapped by measuring the transfer characteristics of the transistors. Based on the performance of the transistors, each wafer was annealed at 165°C under nitrogen for durations ranging between 6 and 13 hours. When required, an additional annealing step at 250°C for durations ranging between 6 and 24 h was performed to increase the bias stress stability of the fabricated thin‐film transistors.

The contour of the arrays was defined using a UV pico‐second pulsed laser (Coherent Aethon picosecond laser system, equipped with a Talisker 500 laser module, Coherent), which was used to cut along the edges of the probes (0.75 W power, 125 mm s^−1^ scan speed, and 4 consecutive laser passes). Before laser‐cutting the samples, a photoresist was spin‐coated to protect the surface from debris generated by the laser ablation. After the cutting, the resist was removed using an NMP‐based stripper for 10 minutes (Microstrip 2001, Fujifilm). Subsequently, a laser lift‐off process was employed to release the probes from the carrier wafer using the same UV laser, by scanning the wafer line‐by‐line from the back side (2.6 W power, 2000 mm s^−1^ scan speed, 2 laser passes, and 50‐µm hatch distance). Finally, the arrays were released by immersing the wafers in water for a few minutes and picked up with tweezers when they started floating.

For connecting the arrays to an external PCB, a Zero Insertion Force (ZIF) connector was employed (Hirose's FH28 series, 0.5‐mm pitch). A stiffener tape was placed under the connector pads of the arrays to increase the thickness, up to ≈250 µm, of the thin PI foil.

### Cross‐Sectional SEM Imaging

Cross‐sectional SEM imaging (Nova 200 NanoLab, FEI) was performed on samples fabricated on glass substrates, without the presence of the polyimide foil. FIB‐SEM (Helios 450 HP, FEI) was used to obtain cross‐sectional images of samples with the full stack, including the polyimide foil.

### Cytotoxicity Assessment of a‐IGZO

Evaluation of cytotoxicity was externalized, and samples were tested by Nelson Labs NV (Leuven, Belgium). Samples consisted of a 24‐nm a‐IGZO layer deposited on a 3‐inch Si wafer. Cytotoxicity was evaluated following ISO 10 993‐5:2009 standard: *Biological evaluation of medical devices – Part 5: Tests for* in vitro *cytotoxicity*, by assessing the biological reactivity of a mammalian monolayer, L929 mouse fibroblast cell culture, in response to extracts of the test sample. Natural rubber and silicone were employed as positive and negative controls, respectively.

For the test, samples were kept under shaking incubation at 37°C for 24 ± 2 h, after which an extract was taken at a ratio of 3 cm^2^ mL^−1^. An unsterile extract was tested. Control articles were autoclaved prior to the preparation of the extract and were extracted under the same conditions as the test sample. The maintenance medium on the cell culture was replaced by the extracts of the test sample or control articles in quadruplicate, and the culture was subsequently incubated for 48 h at 37°C in a humidified atmosphere containing 5% carbon dioxide. Cell viability was quantified through the XTT cell proliferation assay. XTT‐reagent was added to the wells and the cultures were incubated for another 3 to 5 h. The assay works based on the principle that healthy cells were able to convert the yellow XTT dye into the orange formazan compound through mitochondrial dehydrogenase enzymes. As a colorimetric assay, biological reactivity was evaluated by a photo spectrometer at 450‐nm wavelength. Samples with a cell viability of 70% or more were considered non‐cytotoxic.

### Transfer Characteristics of Individual TFTs

Transfer characteristics (I_DS_ – V_GS_ curves) of individual TFTs were measured using a parameter analyzer (Agilent 4156, Agilent Technologies).

### Electrochemical Impedance Spectroscopy

Electrochemical impedance spectroscopy was performed using a 3‐electrode configuration, using a PalmSens4 potentiostat (PalmSens). A platinum wire was used as a counter electrode and a Ag/AgCl electrode served as the reference. All electrodes were tested in 1X PBS buffer at room temperature, and within a Faraday cage to minimize external noise. Complex impedance was measured usually from 1 Hz to 100 kHz at a 70 mV RMS bias.

### Modeling Electrode Impedance Spectra

Electrochemical impedance spectroscopy measurements for electrodes with different electrode diameters (with and without the presence of a thin‐film transistor) were performed from 1 Hz to 100 kHz and fitted using a variation of the Randles circuit model. The fitting was done using PSTrace 5 software (PalmSens), which uses the Levenberg‐Marquardt algorithm for minimization.

### Electrode DC Offset

EDO was characterized by measuring the open circuit potential of the electrodes in 1X PBS buffer against an Ag/AgCl reference electrode using a PalmSens4 potentiostat (PalmSens) (input impedance: > 1 TΩ // 10 pF). After an initial stabilization period of several minutes, multiplexed EDOs of an 8 × 8 array were measured by capturing the open circuit potential at each data line (2 kS s^−1^ sampling rate) while switching between electrodes at a frequency of 1 kHz (125 Hz per select line, 1/8 duty cycle).

### In Vitro Characterization of µECoG Arrays in Addressing Mode of Operation

Characterization was done using probes formed by two subarrays of electrodes separated in space. Each subarray was placed in separate solutions, allowing them to receive distinct sine‐wave inputs applied through platinum wires. Select lines controlling the gate of the select transistors were generated and controlled using a microcontroller (Arduino Uno, Arduino), and a gate driver (UCC21521, Texas Instruments) was used as a level shifter to generate the required voltage levels needed to drive the TFTs (V_sel ON_  =  +5 V, V_sel OFF_  =  −2 V). Recordings from the subarrays were done using standard electrophysiology acquisition systems (RHD2132, Intan Technologies). The headstage filtered (analog bandpass filter: 1 Hz – 500 Hz) and digitalized the signals at a 1 kHz sampling rate. Data from each of the subarrays was recorded sequentially in time, with the recording being paused when switching between electrodes. The recordings were restarted after a brief pause of a couple of seconds, which was necessary for the system to stabilize. All electrodes were tested in 1X PBS buffer at room temperature.

### In Vitro Characterization of µECoG Arrays in Time‐Division Multiplexing Mode of Operation

Characterization was done using a 16 × 16 multiplexed array combined with the dedicated silicon ROIC (0.8 V/0.6 V analog/digital power supply), directly wire‐bonded on a daughterboard. The daughterboard was connected to a motherboard, supplying all the required signals for the operation of the ROIC. The motherboard was controlled through a National Instruments’ acquisition system, formed by an Intel Core i7 embedded controller for PXI Express systems (PXIe‐8135, National Instruments) and a Digital Waveform Instrument (PXIe‐6544, National Instruments). Select signals controlling the gate of the select transistors were generated by the silicon ROIC, but an additional power‐management unit driving the TFTs was implemented off‐chip to accommodate for the higher voltage levels needed by the TFTs (V_sel ON_  =  +5 V, V_sel OFF_  =  ‐2 V). Single‐ended sine‐wave inputs were generated using a signal generation (PXI‐4461, National Instruments) and applied to the test solution through a platinum wire. Recorded signals were captured at a 16 kHz sampling rate per readout super‐channel, or at an effective 1 kHz sampling rate per recording electrode. All electrodes were tested in 1X PBS buffer at room temperature.

The noise of the complete system operating in time‐division multiplexing mode, with the TFTs switching, was measured in saline with the solution grounded through the platinum wire. Data was recorded for 10 s, and the noise was calculated in the frequency domain by integrating the power spectral density of the recorded signal within the bandwidth of interest (1 to 500 Hz). To achieve this, the recorded signals were initially filtered using a first‐order Butterworth high‐pass filter with a corner frequency of 1 Hz to eliminate the residual electrode DC offsets. The power spectral density was then estimated utilizing MATLAB's “pwelch” function, employing a Hanning window without any overlap. The system's noise voltage was computed as the square root of the integrated power spectral density within the frequency range of 1 to 500 Hz. The noise of the ROIC without the array was measured by grounding its inputs and estimated in the same manner. The contribution of the active µECoG array alone was calculated by subtracting the noise power of the ROIC from the overall system's noise.

### Surgical Procedure for Cortex Exposure and Head‐Post Implantation

Approval of all ethical and experimental procedures and protocols was granted by the Animal Ethics Committee, KU Leuven (project license number 089/2021), and performed in line with the National Guidelines on the use of laboratory animals under the Belgian Royal Decree of 29 May 2013 and the European Union Directive for Animal Experiments under Approval No. 2010/63/EU.

C57BL/6 adult mice (≈10 weeks old) were used to validate in vivo the functionality of the developed µECoG arrays. The surgical procedure for cortex exposure and headpost implantation was partially based on a reported protocol for whole‐brain functional ultrasound imaging.^[^ ^40^ ^]^

Anesthesia was induced with an intraperitoneal (IP) injection of ketamine (75 mg kg^−1^ IP) and medetomidine (1 mg kg^−1^ IP) and maintained with booster shots of half the initial dose, administered along the surgery. Before starting the surgery, the absence pedal reflex was checked by pinching the posterior paw of the animal. Buprenorphine (0.1 mg kg^−1^ IP) was given pre‐operatively as analgesic and eye ointment was applied to protect the eyes from drying (Duratears, Novartis). The animal was mounted and immobilized on a stereotactic frame and kept on a heating pad to preserve a stable body temperature. As local anesthesia, bupivacaine (Marcaine) was injected subcutaneously at the site of surgery (50 µL) and left to act for 10 min before incision. The surgical area was shaved and disinfected (Iso‐Betadine, Meda Pharma), and an incision was made in the skin on top of the surgical site with a stainless‐steel surgical blade. The skin was cut over the entire dorsal skull, and collagenous tissue was cleared from the top of the skull using the blade. The left temporal muscles were retracted and treated with tissue adhesive (Vetbond, 3 M). A stainless‐steel bone screw was placed over the cerebellum or in the frontal bone, to be used as a ground electrode during recordings. The head‐post was placed and fixed to the skull with dental cement (Superbond C&B, Prestige Dental). The size of the craniotomy was determined by the area of the µECoG array to be tested. Larger arrays required a bilateral craniotomy (between bregma +3.00 to −7.00 mm, and 5.00 mm apart from the sagittal suture), covering a big part of the cortical surface. The skull was gently thinned over the region of interest with a low‐speed dental drill and constantly perfused with cold saline to avoid overheating of the brain. Once the skull was flexible and vessels from the dura were visible through the wet bone, the skull was carefully peeled off without damaging the dura using fine‐tip forceps. In case of bleeding, a sterile compressed sponge was applied to stop the outflow.

### Acute Electrophysiological Recordings

The functionality of the arrays was validated through electrophysiological recordings in mice. The spontaneous activity as well as somatosensory evoked potentials were recorded in response to peripheral electrical stimulation of the animal's paws. Surgery and recordings were performed in a single session, which lasted around 5 to 6 hours. In this case, the procedure corresponded to a terminal experiment.

The anesthetized animal was head‐fixed and placed on an antivibration table, inside a Faraday cage to minimize external sources of interference. The animal was kept under a heating gel pad (Deltaphase Isothermal Pads, Braintree Scientific) to preserve a stable body temperature. Electric heating pads were avoided, to minimize the coupling of electrical noise into the recordings. The developed µECoG array was placed epidurally over the cortex, including the region of interest, i.e., the primary somatosensory cortex (S1). During the experiments, the exposed brain was kept hydrated by administering saline on the cranial window in order to prevent toughening of the dura mater. For all recordings, headstages were grounded to the implanted ground screw, and measurements were referenced to ground.

Recordings from arrays in the addressing mode were performed using a commercial electrophysiological acquisition system (Open Ephys Acquisition Board^[^ ^41^ ^]^ connected to Intan's RHD2132 32‐channel amplifier headstage). The µECoG arrays were connected to the headstage, where signals were filtered (analog bandpass filter: 1 Hz – 2 kHz), amplified, and digitalized at a 10 kHz sampling rate. Digital select lines for addressing the thin‐film transistors in the arrays were generated using the digital outputs of the Open Ephys acquisition board, after modification of the board firmware. The amplitude of generated digital signals was shifted from [0 V to +5 V] to [−2 V to +5 V] using a gate driver (UCC21521ADW, Texas Instruments), in order to ensure correct voltage levels for a proper switching of the transistors in the array.

Recordings from arrays in time‐division multiplexing mode were performed using the dedicated readout integrated circuit capable of handling multiplexed recordings.^[^ ^26^ ^]^ The arrays were connected to a headstage containing the ROIC, where signals were amplified and digitalized at a 16 kHz sampling rate per readout super‐channel, or at an effective 1 kHz sampling rate per recording electrode. The headstage was connected to a motherboard, supplying all the required signals for the operation of the ROIC. A National Instruments PXI‐6544 was used to program the chip and collect the digital output data.

In each recording session, a baseline period of 2 min was recorded, corresponding to the resting state. Peripheral electrical stimulation of different paws was done by injecting current pulses (DS3 Isolated Current Stimulator, Digitimer) through two thin‐needle electrodes made from acupuncture needles, inserted under the skin of the selected paws. Stimulating electrodes were placed in the inner side of the paw, between the footpad and wrist. Current stimulation was employed, as it allows control over the amount of injected charge. The current intensity was adjusted by injecting individual current pulses of increasing amplitude (200 µA to 1 mA) and selecting the minimum intensity capable of eliciting a weak paw twitching reflex. Each stimulation trial consisted of 10 consecutive monophasic square‐wave current pulses of 2‐ms duration, repeated over 10 cycles with a frequency of 1 Hz or 1.5 Hz, and with an inter‐cycle interval of 5 seconds.

Recording sessions lasted for 1 to 3 h. Sterile saline at room temperature was injected subcutaneously every hour to prevent animal dehydration (0.3 mL). After concluding the recording session, animals were euthanized using carbon dioxide.

### Signal Processing of Recorded Data

All signal analysis was done using MATLAB (MathWorks). Recorded data was bandpass filtered using a digital first‐order Butterworth filter between 1 Hz and 500 Hz. Recordings with the ROIC in time‐division multiplexing mode presented sporadic glitches that caused DC shifts in the recorded signal, resulting from the update of the EDO cancellation loop in the ROIC. These glitches were removed offline, by stitching the recorded signal whenever a glitch occurred.

To better visualize the captured physiological oscillations, recorded signals were filtered in the delta (1 Hz–4 Hz), spindle (10 Hz–16 Hz), and ripple (100 Hz–150 Hz) frequency bands using a second‐order Butterworth filter. Spectrograms were calculated in the frequency ranges [1 to 55 Hz] and [50 to 500 Hz] using MATLAB's spectrogram function and employing a Hamming window of 1024 samples and 90% overlap, and one of 512 samples and 90% overlap, respectively. Color maps reflecting the spatiotemporal dynamics of recorded activity were calculated from the mean voltage calculated in 5 ms or 50 ms time bins. The RMS instantaneous power in the different frequency bands was calculated from a 2‐minute‐long recording by calculating the RMS of the absolute value of the Hilbert transformed signal (analytic signal).

Cortical ripples were detected and quantified using the Free Moving Animal (http://fmatoolbox.sourceforge.net) toolbox and its sequential detection algorithm. First, the signal was bandpass‐filtered between 100 – 200 Hz (third‐order Butterworth) and rectified. Events were identified as the periods in which the filtered envelope was one time higher than the standard deviation (SD) of filtered traces, and for which the peak envelope was 2.5 times higher than SD. Consecutive events separated by less than 30 ms were merged, and those with a duration outside the 20 ms to 400 ms range were discarded.

For recordings including electrical stimulation of one of the paws, averaging of the response after stimulation was based on the alignment to the TTL pulse that triggered the electrical stimulation. The RMS instantaneous power after stimulation was calculated from the averaged responses filtered in the frequency ranges between 30 Hz and 500 Hz using a second‐order Butterworth filter, and it was done by calculating the RMS of the absolute value of the Hilbert transformed signal in a 10 to 50 ms time window after stimulation.

### Ethics Approval Statement

Approval of all ethical and experimental procedures and protocols was granted by the Animal Ethics Committee, KU Leuven (project license number 089/2021), and performed in line with the National Guidelines on the use of laboratory animals under the Belgian Royal Decree of 29 May 2013 and the European Union Directive for Animal Experiments under Approval No. 2010/63/EU.

## Conflict of Interest

The authors declare no conflict of interest.

## Supporting information

Supporting Information

## Data Availability

The data that support the findings of this study are available from the corresponding author upon reasonable request.
