# Supplementary material for: Multiplexed Surface Electrode Arrays Based on Metal Oxide Thin‐Film Electronics for High‐Resolution Cortical Mapping
Source: Adv Sci (Weinh). 2023 Dec 25;11(10):2308507. doi: 10.1002/advs.202308507 (PMC10933637; doi:10.1002/advs.202308507)
Supplement: Supplementary file 1 — Supporting Information [file ADVS-11-2308507-s001.pdf]

ADVANCED SCIENCE
Open Access

Supporting Information

for *Adv. Sci.*, DOI 10.1002/advs.202308507

Multiplexed Surface Electrode Arrays Based on Metal Oxide Thin-Film Electronics for High-Resolution Cortical Mapping

Horacio Londoño-Ramírez, Xiaohua Huang, Jordi Cools, Anna Chrzanowska, Clément Brunner, Marco Ballini, Luis Hoffman, Soeren Steudel, Cédric Rolin, Carolina Mora Lopez, Jan Genoe and Sebastian Haesler**

Supporting Information

Multiplexed Surface Electrode Arrays Based on Metal Oxide Thin-Film Electronics for High-Resolution Cortical Mapping

Horacio Londoño-Ramírez, Xiaohua Huang, Jordi Cools, Anna Chrzanowska, Clément Brunner, Marco Ballini, Luis Hoffman, Soeren Steudel, Cédric Rolin, Carolina Mora Lopez, Jan Genoe, Sebastian Haesler**

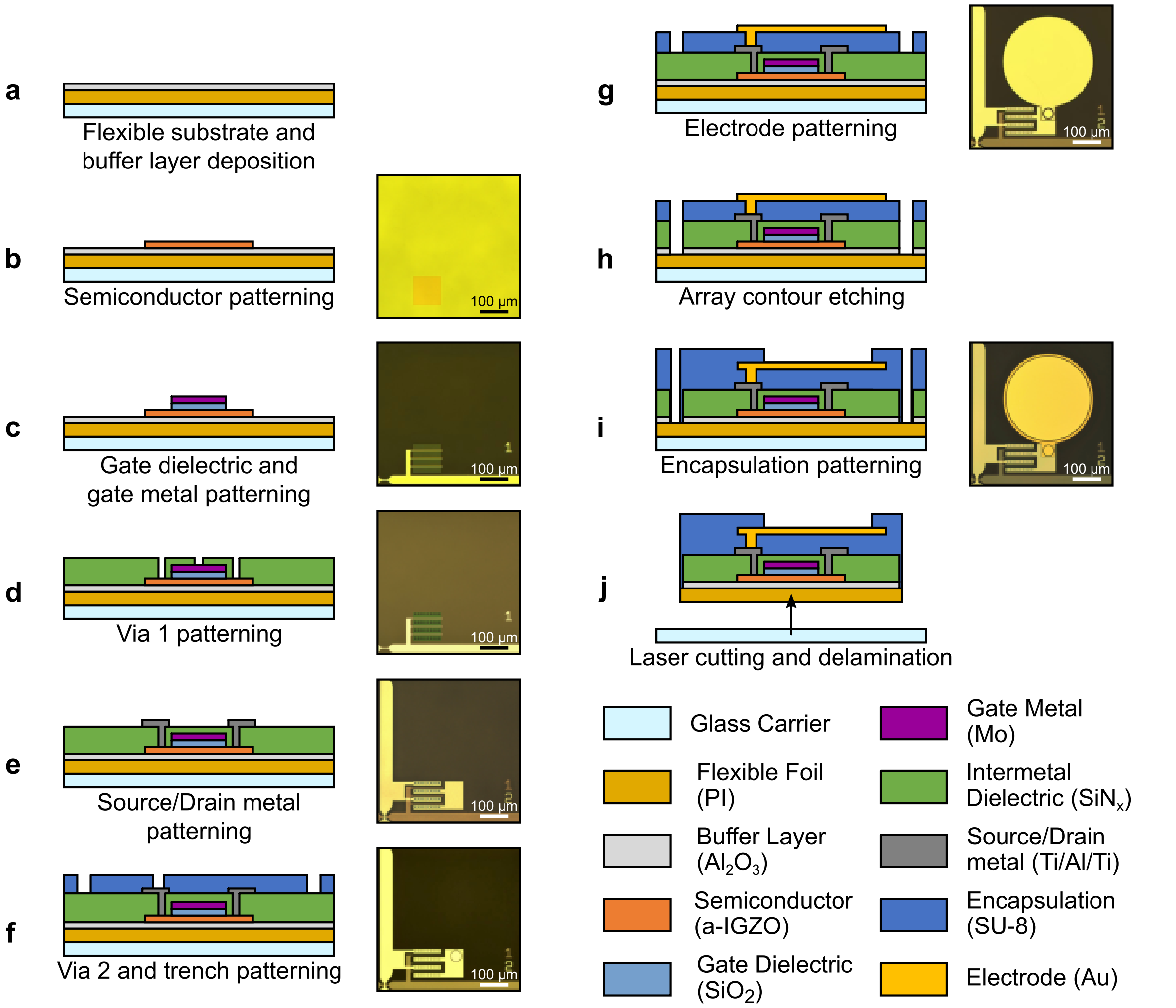

Figure S1. Schematic cross-section view and microphotographs of the main process steps of the fabrication of the µECoG array based on a-IGZO thin-film transistors. The fabrication steps are as follows: a) Flexible substrate and buffer layer deposition, b) Semiconductor deposition and patterning, c) Gate dielectric and gate metal deposition and patterning, d) Intermetal dielectric deposition and Via 1 patterning, e) Source/Drain metal deposition and patterning, f) Dielectric deposition and Via 2 patterning, g) Electrode deposition and patterning, h) Array contour etching, i) Encapsulation deposition and patterning, j) Laser cutting and delamination.

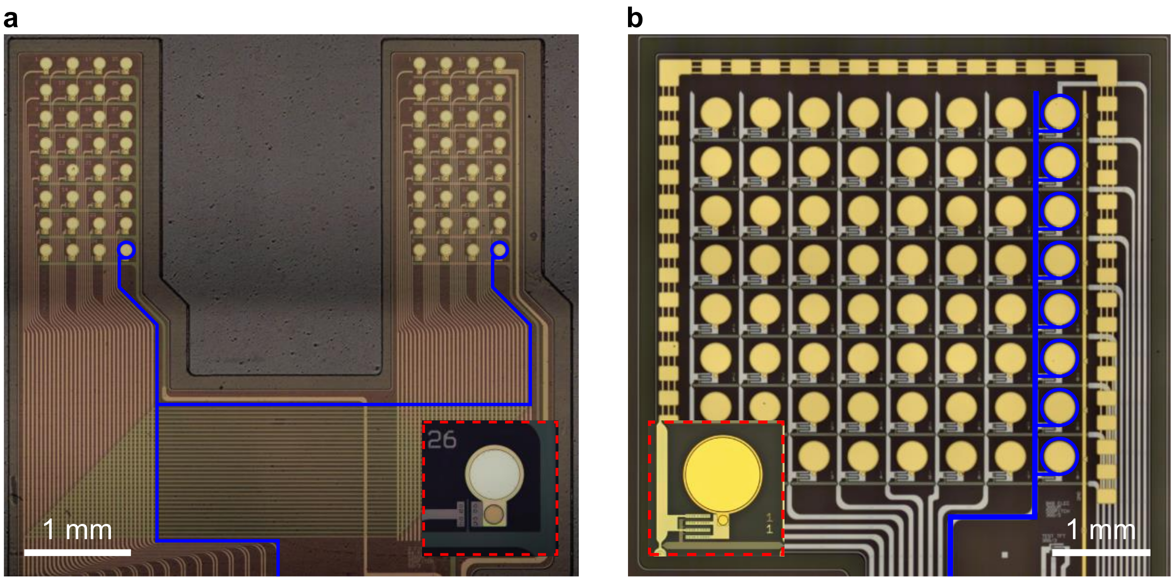

Figure S2. µECoG arrays based on a-IGZO thin-film transistors with different electrode configurations. a) Microphotograph of a 62-channel µECoG array released from the glass carrier wafer. The array is formed by 2 subarrays spaced 2 mm apart, with each subarray containing 31 electrodes (including an electrode without a TFT) arranged in an 8×4 matrix: 100-µm electrode diameter, 250-µm electrode pitch, transistor dimensions W/L = 50 µm/3 µm, and ~2.2×1.5 mm^2 array dimensions. b) Microphotograph of a 64-channel µECoG array on the carrier wafer: 8×8 pixels, 300-µm electrode diameter, 500-µm electrode pitch, reference electrode surrounding the array, transistor dimensions W/L = 300 µm/3 µm, and ~5.4×5.4 mm^2 array dimensions. Insets in each figure show a magnification of the corresponding pixel. For each figure, groups of electrodes sharing a single data line are highlighted in blue.

Table S1. Biocompatibility classification of the constituent materials of the µECoG arrays based on a-IGZO thin-film transistors.

Material	Biocompatibility	Reference
**Metals**		
Au	Nontoxic	[1–3]
Mo	Nontoxic	[1,4,5]
Ti	Nontoxic	[6,7]
Al	Al^{3+}: Neurotoxic at high concentrations	[1,5,8]
**Dielectrics**		
Polyimide	Nontoxic	[9,10]
SU-8	Nontoxic	[3,7,11]
SiO_2	Nontoxic	[1,3,7]
SiN_x	Nontoxic	[3,7]
Al_2O_3	Nontoxic	[1,12,13]
**Semiconductor and constituent metal oxides**		
a-IGZO	Nontoxic (ISO 10993-5:2009)	This work, [14]

Table S2. Cytotoxicity assessment of a-IGZO. Cell viability after 48-hours exposure to extracts from samples with a-IGZO, positive control (natural rubber), and negative control (silicone). Cytotoxicity was evaluated according to ISO 10993-5:2009 standard: *Biological evaluation of medical devices - Part 5: Tests for in vitro cytotoxicity*. Samples with a cell viability lower than 70% are considered cytotoxic.

Sample	Cell Viability	Assessment
Positive Control	3%	Cytotoxic
Negative Control	107%	Non-cytotoxic
a-IGZO	96%	Non-cytotoxic

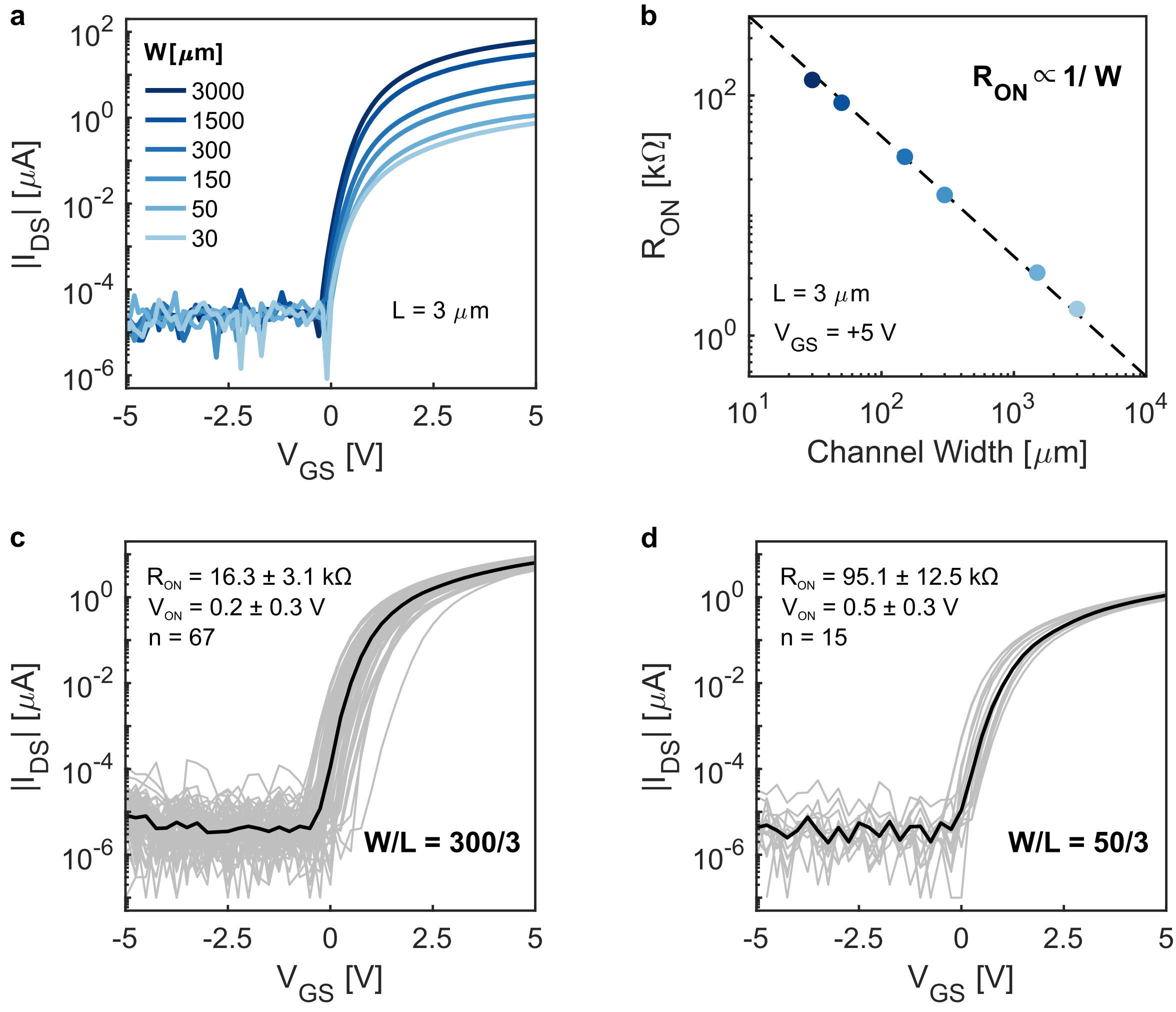

Figure S3. Electrical characterization of a-IGZO thin-film transistors working as select transistors in the µECoG arrays. a) Transfer characteristics (I_{DS} vs V_{GS}) and b) calculated ON resistance for TFTs with different channel widths (W = 30 µm to 3,000 µm) and fixed channel length (L = 3 µm). c) Free-standing TFTs with dimensions W/L = 300 µm/3 µm (n = 67) and d) W/L = 50 µm/3 µm (n = 15). For all measurements, V_{DS} was 100 mV. R_{ON} is calculated at V_{GS} = +5V, and V_{ON} as the gate voltage for which I_{DS} = 1 nA. For c) and d), gray traces correspond to single devices while thicker black traces correspond to median values.

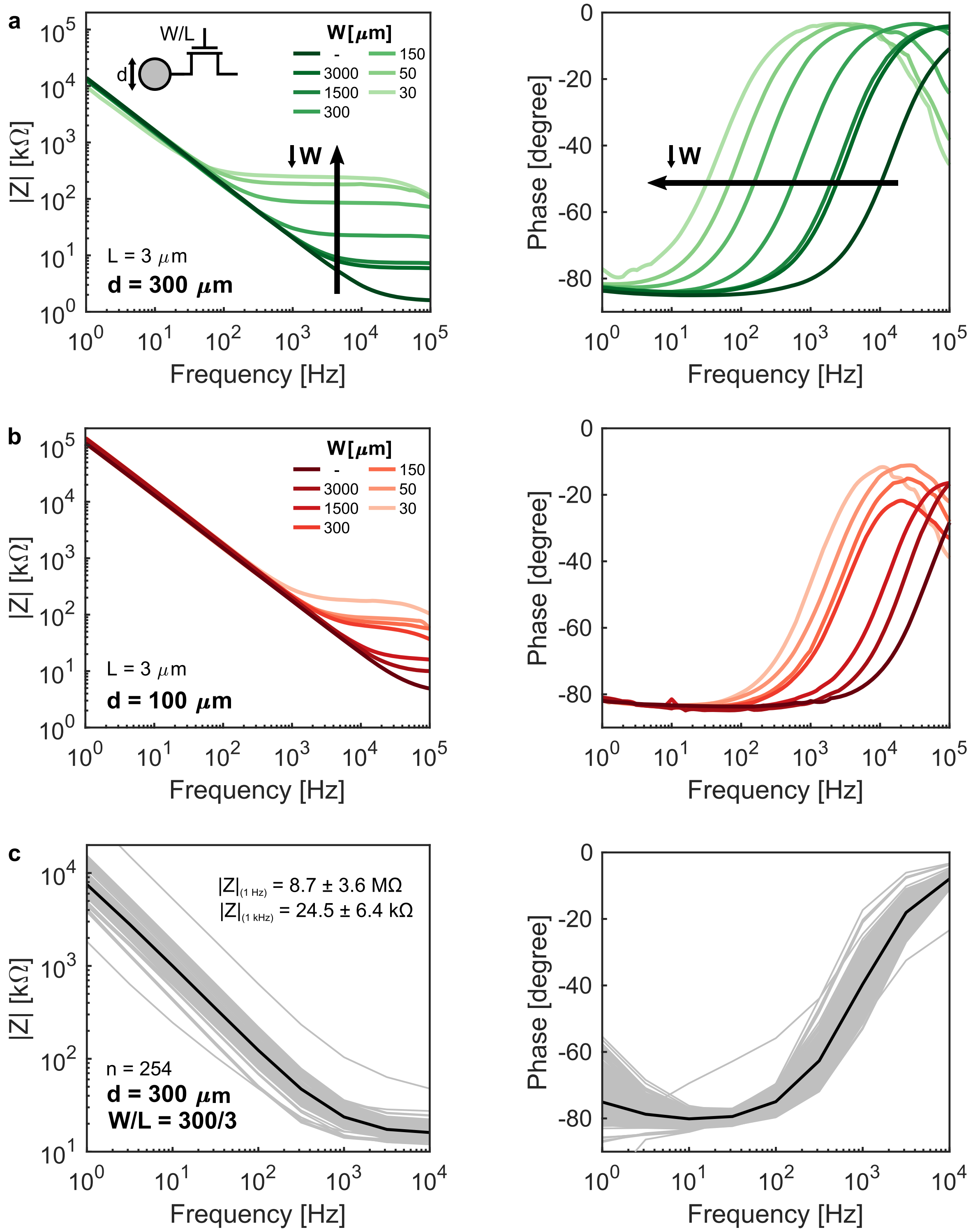

Figure S4. Characterization of recording pixels for different transistor and electrode sizes. Impedance spectra of various pixel designs formed by gold electrodes with and without the presence of a select transistor for two electrode diameters, a) 300-μm and b) 100-μm electrodes,

connected to transistors with varying channel widths (W = 30 µm to 3,000 µm) and fixed channel length (L = 3 µm). The effect of adding a TFT is seen as an increase of the impedance at higher frequencies, equivalent to the ON resistance of the transistor. c) Impedance spectra of all the pixels of a 16×16 µECoG array (300-µm electrode diameter, and transistor dimensions of W/L = 300 µm/3 µm, n = 254, data from two defective pixels is excluded). Gray traces correspond to single pixels while thicker black traces correspond to median values.

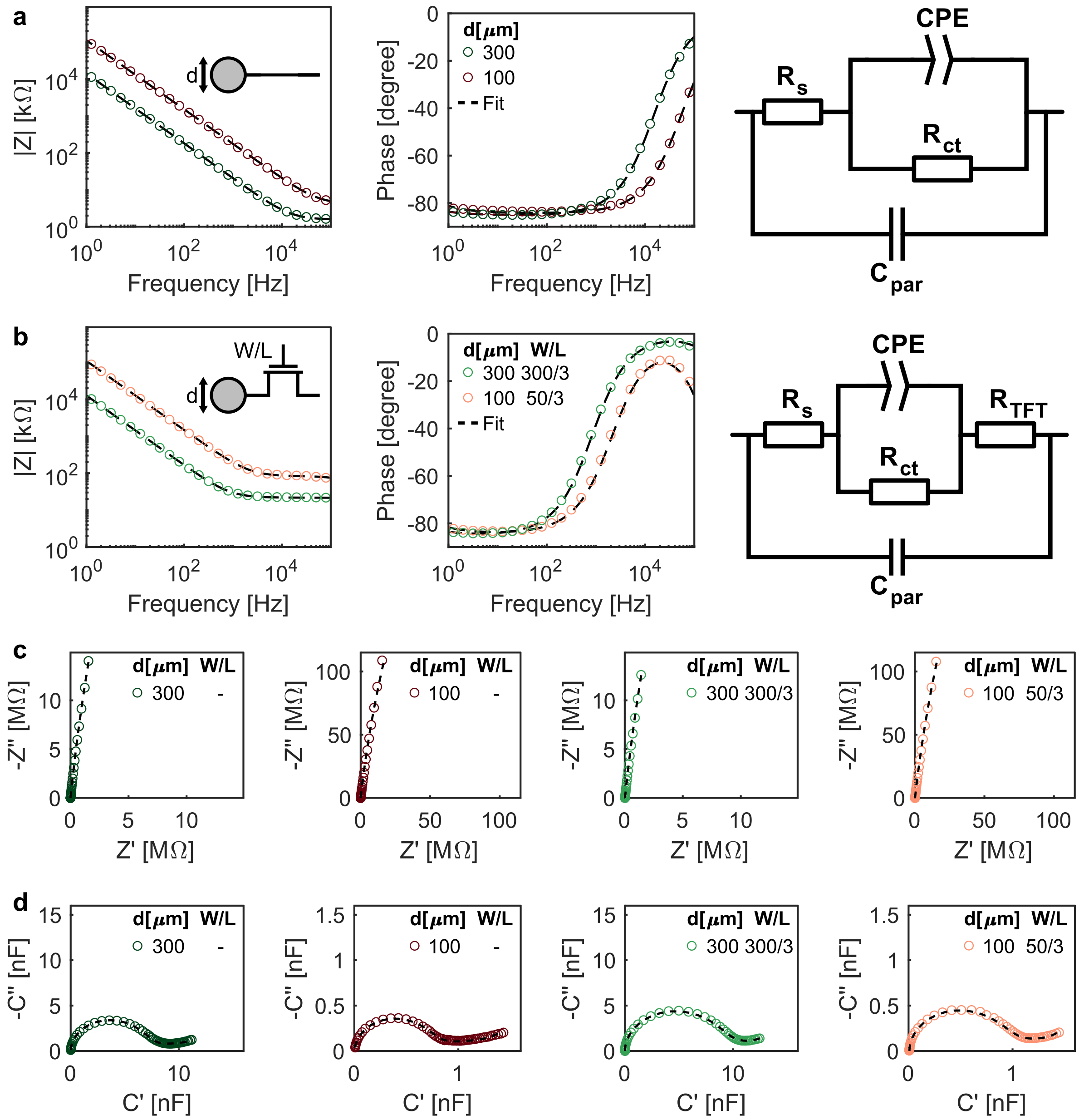

Figure S5. Modeling impedance spectra of recording pixels. a) Bode plot (magnitude and phase) of the electrochemical impedance spectroscopy data and corresponding fit of pixels formed by just a gold electrode, and b) of pixels formed by a gold electrode and a select transistor. For each condition, data from two different electrode diameters is presented (300 µm and 100 µm). On the right side, the equivalent electrical circuit models used for fitting the impedance spectra are presented. The simplified Randles circuit model was employed with the addition of a capacitance in parallel (C_{par}) accounting for parasitic capacitances, and a resistor in series (R_{TFT}) accounting for the R_{ON} of the TFT, in the corresponding case. c) Nyquist plot:

real against imaginary part of the impedance, and d) Nyquist plot of the complex capacitance: real against imaginary part of the capacitance (defined as C = 1/jωZ), and corresponding fits for the four pixels presented in a) and b).

Table S3. Calculated values of the individual electrochemical components used to model the different recording pixels: gold electrodes (300 µm and 100 µm in diameter) with and without the presence of a select transistor (W/L = 300 µm/3 µm and W/L = 50 µm/3 µm). The calculated average error of the fit is included. The employed equivalent circuit models are presented in Figure S5. The impedance of the constant phase element (CPE) is defined as $Z_{CPE} = \frac{1}{(j\omega)^{n_{CPE}} \cdot Q_{CPE}}$, where j is the imaginary unit, ω is the angular frequency, and Q_{CPE} and n_{CPE} ($0 < n < 1$) are frequency independent terms.

	**300 µm (No TFT)**	**300 µm W/L = 300/3**	**100 µm (No TFT)**	**100 µm W/L = 50/3**
$R_{Total} = R_s + R_{TFT}$	1.6 kΩ	21.7 kΩ	4.1 kΩ	83.4 kΩ
Q_{CPE}	12.5 nF·s^{n-1}	13.9 nF·s^{n-1}	1.6 nF·s^{n-1}	1.6 nF·s^{n-1}
n_{CPE}	0.95	0.95	0.94	0.94
R_{ct}	535.8 MΩ	517.7 MΩ	2.1 GΩ	2.4 GΩ
C_{par}	6.6 pF	5.9 pF	5.4 pF	8.8 pF
$R_{TFT} = R_{Total} – R_{s\,(No\,TFT)}$	-	~ 20 kΩ	-	~ 80 kΩ
$\chi 2$	$3\cdot10^{-4}$	$10\cdot10^{-4}$	$2\cdot10^{-4}$	$30\cdot10^{-4}$
$\sqrt{\chi 2}$: **Average error of the fit**	1.7 %	3.2 %	1.4 %	5.5 %

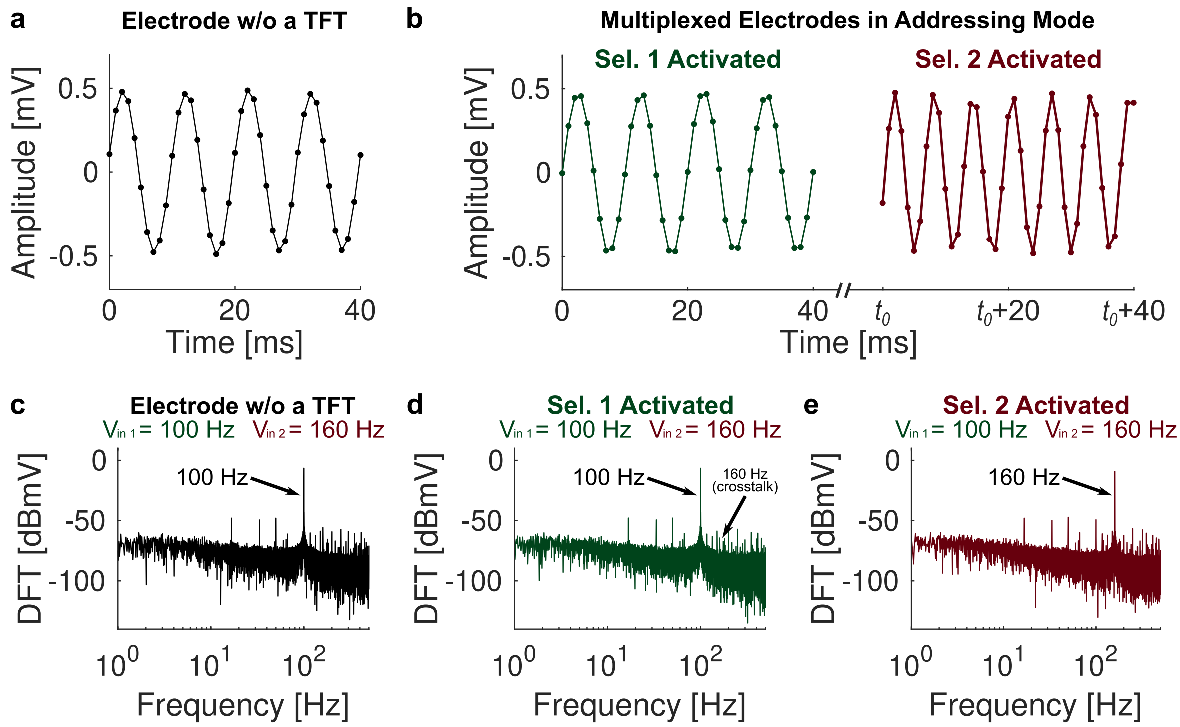

Figure S6. *In vitro* recordings in addressing mode from a µECoG array formed by two subarrays of electrodes (100-µm electrode diameter, and transistor dimensions of W/L = 50 µm/3 µm). The subarrays are placed in separate solutions, receiving distinct sinusoidal input signals ($V_{in\,1}$ = 1 mV_{pp}, 100 Hz, and $V_{in\,2}$ = 1 mV_{pp}, 160 Hz). a) Recorded signal from an electrode without a TFT present in one of the subarrays, and b) from two addressable electrodes located in different subarrays but sharing a single data line. Data from each electrode was recorded sequentially in time using a standard electrophysiology acquisition system. The recording was paused for a short time (t_0) while switching between electrodes. c) - e) Discrete Fourier Transform (DFT) of time-domain data in a) and b).

Aliasing of Out-of-Band Noise in Time-Division Multiplexed Recordings

In time-multiplexed recordings, input channels are sampled sequentially in time, at a per-channel sampling frequency f_{ch}. Consequently, the overall sampling frequency (f_s) must be increased compared to non-multiplexed recordings to allow the acquisition of data from all multiplexed channels. In other words, the acquisition bandwidth ($f_s/2$) must be larger than the signal band ($BW_{Sig.}$) by at least a factor equal to the multiplexing ratio (N). This can be summarized in the following expression:

$$f_s/2 = N \cdot f_{ch}/2 \geq \mathrm{N} \cdot BW_{Sig.} \tag{1}$$

Additionally, increasing the acquisition bandwidth results in an equivalent rise in the noise bandwidth (BW_n). Following the data acquisition, the neural signals captured by each electrode are reconstructed during the demultiplexing step by subsampling the time-multiplexed signal at the specific per-channel sampling rate. The demultiplexing process causes noise above the signal band to fold back into the band of interest (**Figure S7**a). This occurs because the Nyquist sampling criterion is not satisfied, as the signals from each channel are subsampled at a frequency lower than twice the noise bandwidth.

The folding of out-of-band noise results in an increase in the total in-band noise power in the final demultiplexed output. The noise power of a non-multiplexed electrode sampled at a frequency f_{ch}, and using an anti-aliasing, filter will be:

$$V_{n(Non\ Mux.)}^2 = \int_0^{\frac{f_{ch}}{2}} S_{v_n}(f)\, df \tag{2}$$

, where $S_{v_n}(f)$ represents the noise power spectral density. In a time-multiplexed system this noise will increase to:

$$V_{n(Mux.)}^2 = \int_0^{\frac{f_s}{2}} S_{v_n}(f)\, df = \int_0^{N \cdot \frac{f_{ch}}{2}} S_{v_n}(f)\, df \tag{3}$$

The exact magnitude of the noise increase will depend on the spectrum of the noise power density, the multiplexing ratio, and the per-channel sampling frequency. The higher the multiplexing ratio or the per-channel sampling frequency, the greater the degradation in the noise levels due to an increase in the noise bandwidth.

Focusing on our µECoG array, its noise levels are dominated by the pixel noise, set by the electrode and the thin-film transistor. The noise power spectral density of the pixel can be estimated from the real part of its impedance as:

$$S_{v_{n(Pixel)}}(f) = 4k_B T \cdot \Re[Z_{pixel}(f)] \quad (4)$$

, where k_B is Boltzmann's constant, T is the temperature in kelvins, and $Z_{pixel}(f)$ the frequency dependent impedance.[15] For our standard pixel size (300-µm electrode diameter, W/L = 300 µm/3 µm), its noise power spectral density can be calculated from the electrode impedance presented in Figure 2b using equation (4) (Figure S7b). Using equation (3), the noise voltage can be estimated for different multiplexing ratios or switching frequencies (**Table S4**). For the complete system, the noise contribution of the recording electronics should be included.

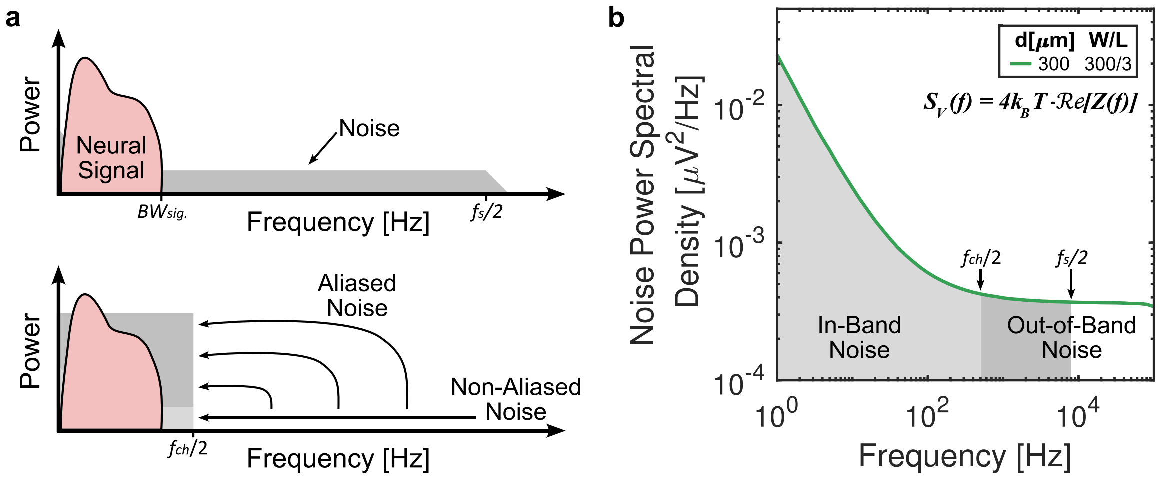

Figure S7. Aliasing of out-of-band noise in time-division multiplexed recordings. a) Frequency domain representation of the noise-folding that occurs during demultiplexing as a result of signal sampling directly at the electrode without an anti-aliasing filter that restricts out-of-band noise. b) Noise power spectral density estimated from the measured pixel impedance, for a gold electrode 300-µm in diameter with a TFT with W/L = 300 µm/3 µm. The in-band noise and the out-of-band noise that will be aliased are highlighted in light and dark gray, respectively, for a per-channel sampling frequency of 1 kHz and a multiplexing ratio of 16. To minimize the noise bandwidth, the recording electronics must filter out the noise above $f_s/2$.

Table S4. Estimated pixel noise contribution for recordings in time-division multiplexing mode. Total noise, including aliasing, is calculated for the standard pixel size (300-µm electrode diameter, W/L = 300 µm/3 µm), with a fix multiplexing ratio of 16, and for different switching frequencies.

Switching Frequency	Noise [µV_{rms}]
No Multiplexing	0.64
500 Hz	1.63
1 kHz	2.25
2 kHz	3.14

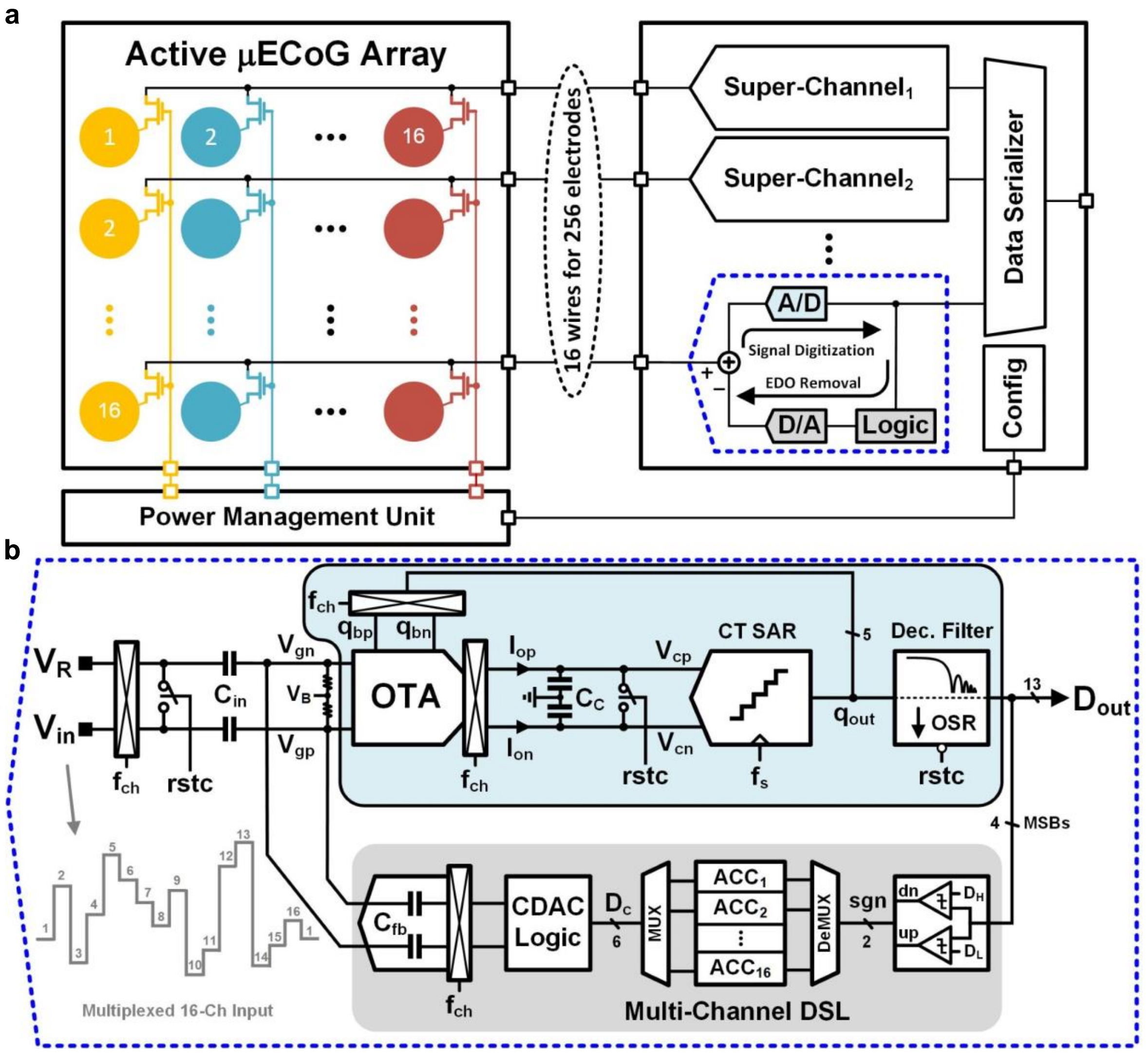

Figure S8. Readout IC for time-division multiplexed recordings (© 2022 IEEE. Reprinted, with permission, from [16]). a) Simplified block diagram of the complete proposed system formed by the µECoG array and the dedicated ROIC. b) Block diagram of the proposed super-channel capable of recording from 16 multiplexed electrodes. For more details regarding the functioning of the ROIC, please refer to our previous publication dedicated to it.[17]

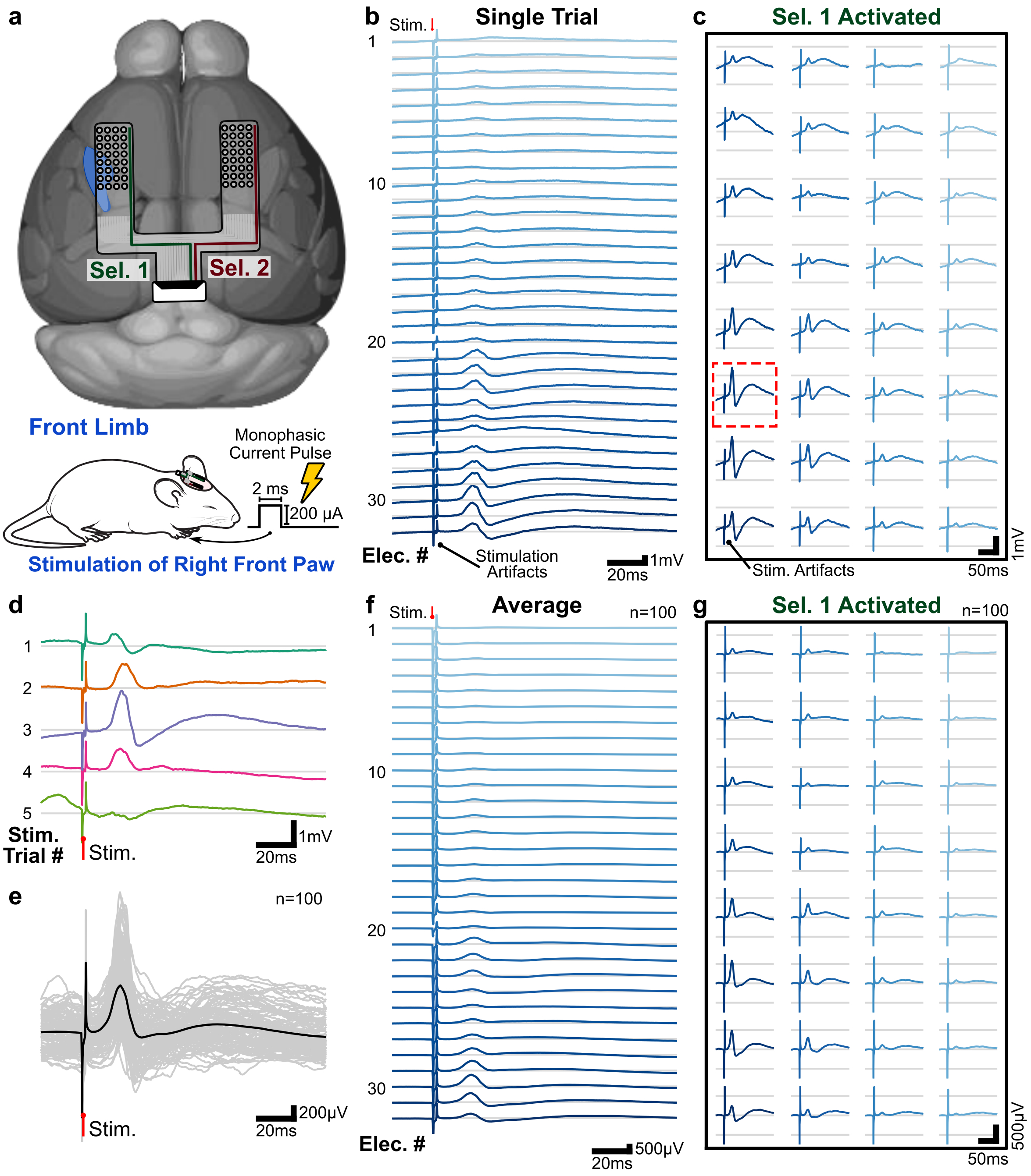

Figure S9. *In vivo* recordings of somatosensory evoked potentials using a μECoG array in addressing mode. a) Representation of the placement of a μECoG array with two subarrays over the cortical surface, with one of the subarrays partially covering the front limb region of the left somatosensory cortex. The front paw of an anesthetized mouse was electrically stimulated using monophasic current pulses (200 μA in amplitude, and 2 ms in duration) to elicit an evoked response. b) Single-trial recorded response from one of the addressed subarrays after electrical

stimulation of the contralateral front paw, and c) spatial distribution of recorded evoked potentials. The electrode with the strongest response is highlighted. d) Difference in the evoked response after successive stimulation trials for a selected electrode. e) Averaged response of a selected electrode (gray: single trials, black: average value, n = 100) and f) - g) of all the electrodes of the addressed subarray.

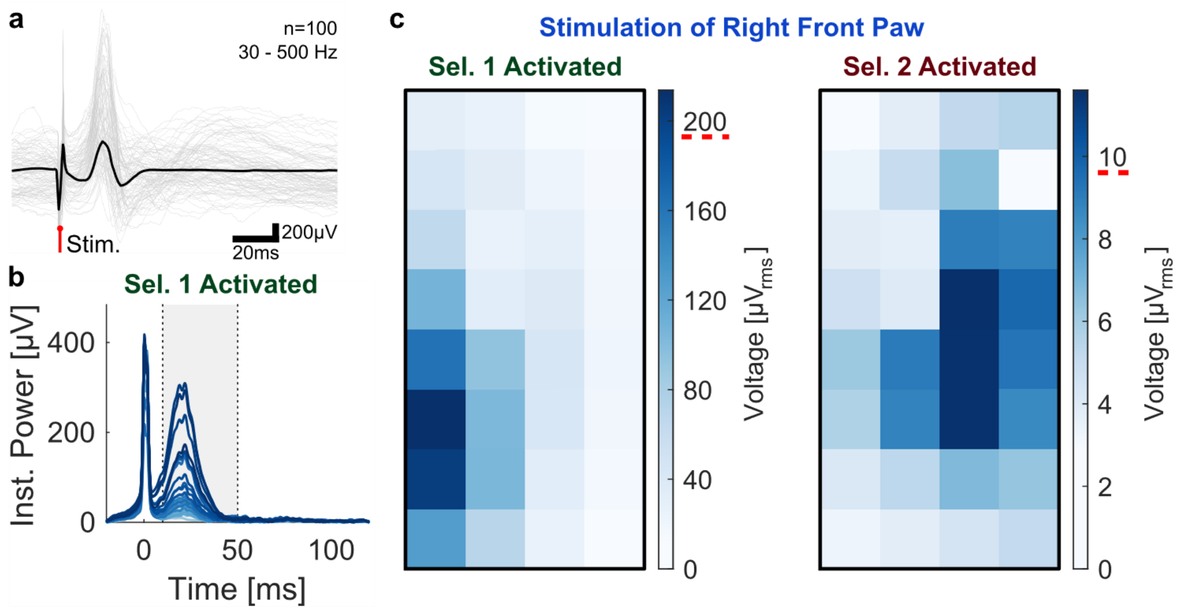

Figure S10. a) Average gamma response of a selected electrode recording SSEPs after electrical stimulation of one of the paws (gray: single trials, black: average value, n = 100). Trials aligned to the stimulation onset (TTL signal). b) Instantaneous power of the averaged responses filtered between 30 Hz and 500 Hz for all electrodes of the subarray located contralaterally to the stimulated paw. The time frame used to calculate the RMS instantaneous power (10 to 50 ms after the stimulation onset) is highlighted. c) Localized gamma response to electrical stimulation of the right front paw, recorded with a µECoG array with two subarrays. The subarray activated with select line #1 was located in the hemisphere contralateral to the stimulated paw, while the subarray activated with select line #2 was in the ipsilateral hemisphere. The color map indicates the RMS instantaneous power in the specified time frame. Different scalebars are used for each subarray.

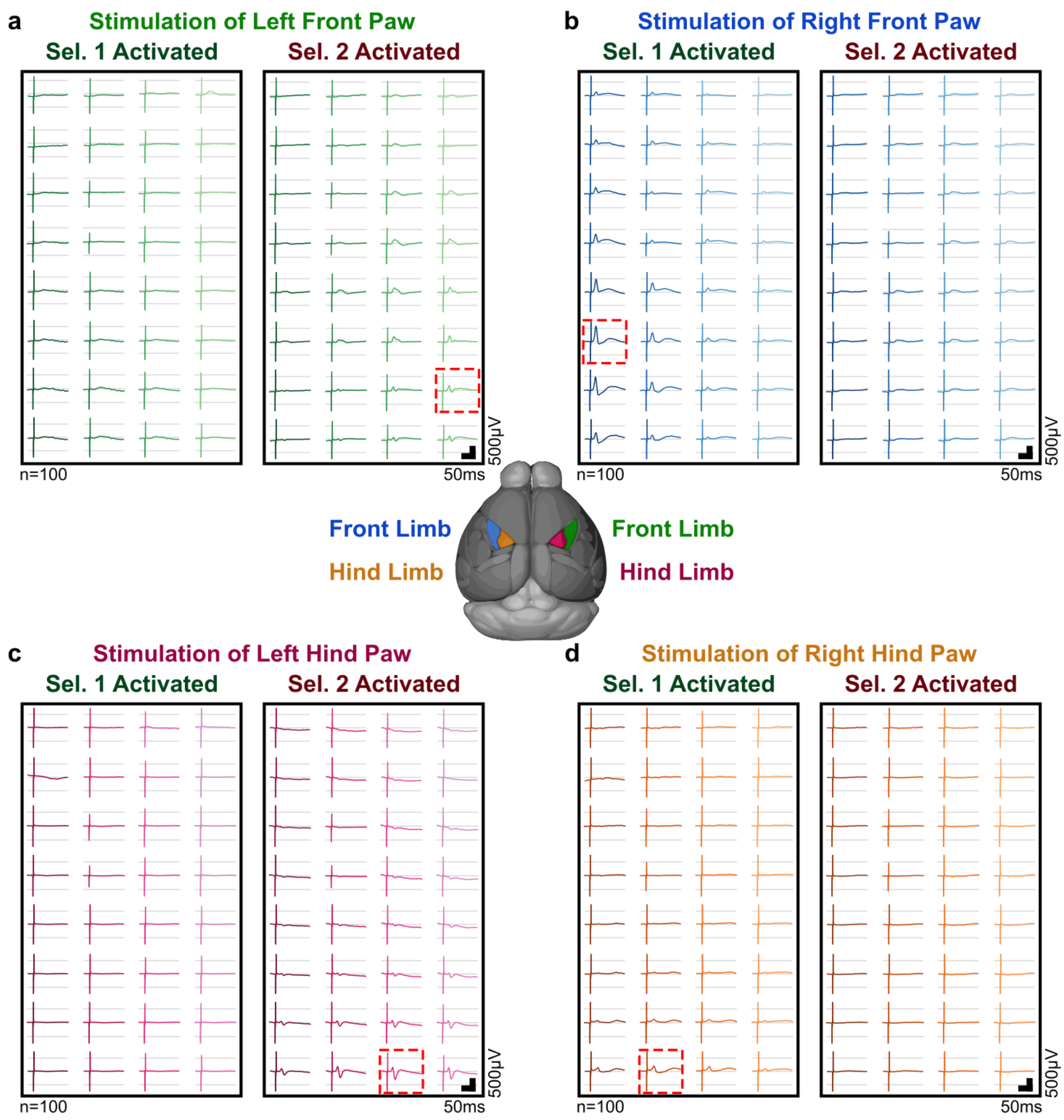

Figure S11. Distinct evoked responses elicited by the electrical stimulation of the four paws of the animal, recorded with a µECoG array with two subarrays, each placed over one of the brain hemispheres (n = 100). Averaged responses elicited after the stimulation of the a) left front paw, b) right front paw, c) left hind paw, and d) right hind paw. In each experiment, the subarrays were recorded sequentially. Electrodes with the strongest response for each stimulation condition are highlighted. Central inset: Location of the somatosensory areas representing the front and hind limbs in the mouse brain.

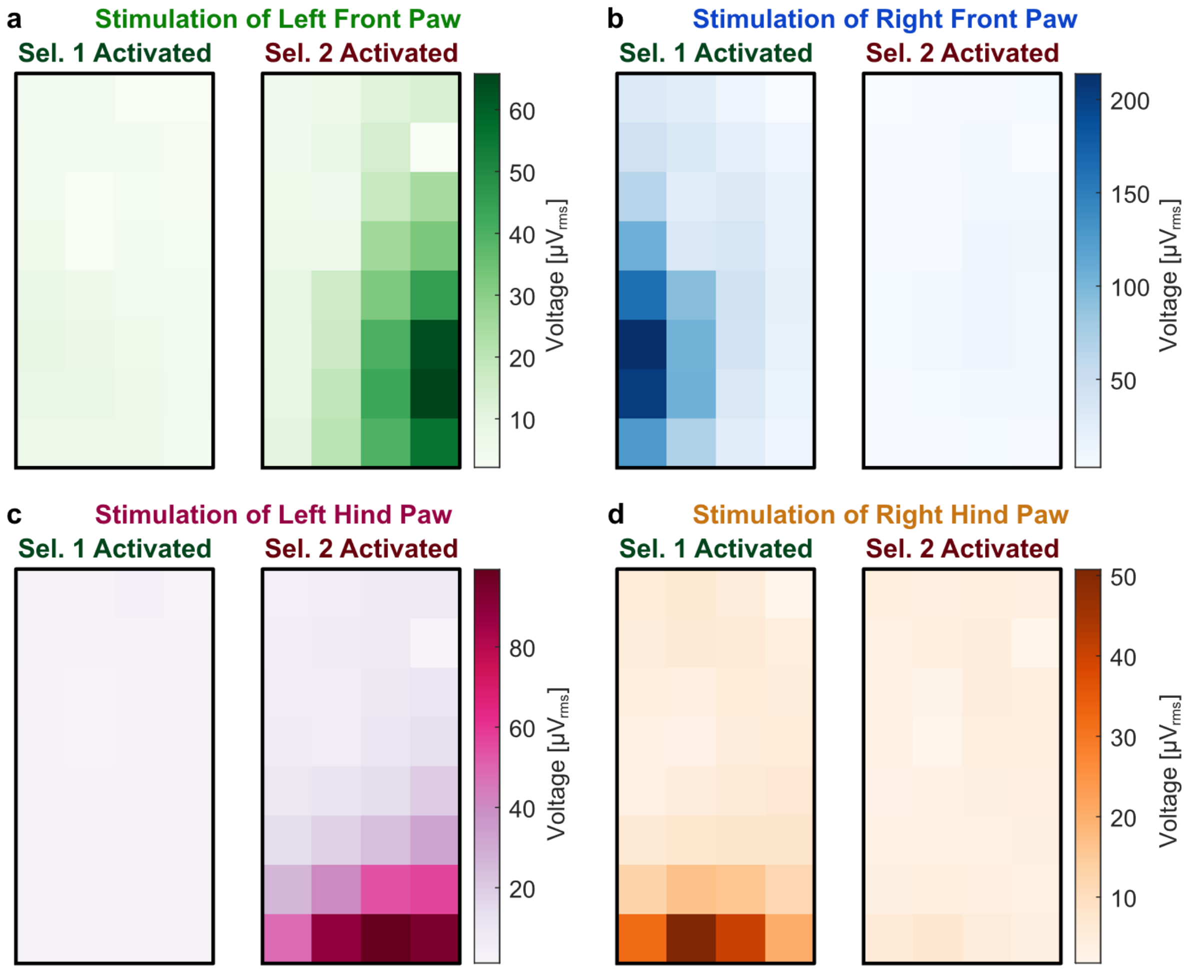

Figure S12. Localized gamma responses to electrical stimulation of the four paws, recorded with a µECoG array with two subarrays (n = 100). Averaged responses for the stimulation of the a) left front paw, b) right front paw, c) left hind paw, and d) right hind paw. The color map indicates the RMS instantaneous power of the measured responses filtered between 30 Hz and 500 Hz. A single scalebar is used for both subarrays under each stimulated paw, but different scalebars are used between stimulated paws.

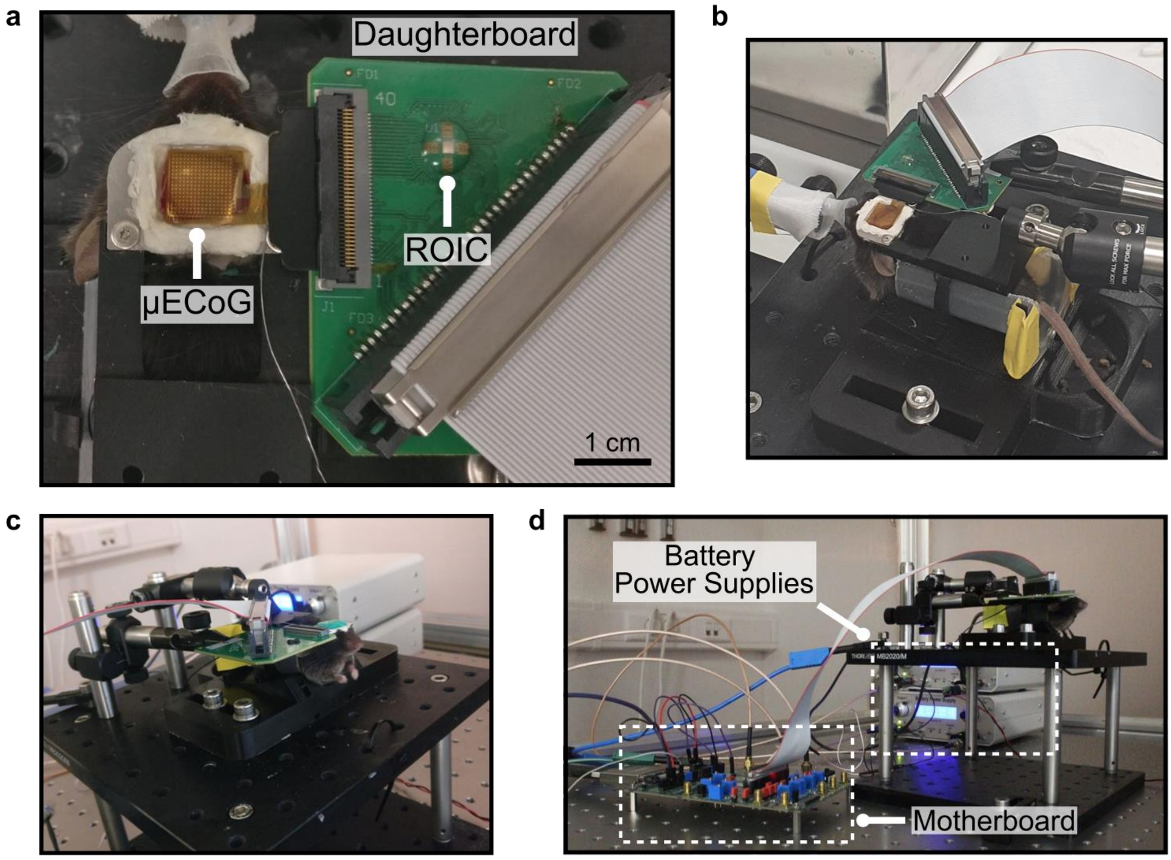

Figure S13. Photographs depicting the experimental setup used for in vivo recordings. a) A 16×16 µECoG array placed over the cortex of an anesthetized mouse. The µECoG array was connected to a daughterboard with the wire bonded silicon ROIC via a Zero Insertion Force connector. b) to d) Photographs showing the implanted probe, the daughterboard, and the complete set up. The daughterboard was connected to a motherboard, which was responsible for supplying all necessary signals for the operation of the ROIC and allowed debugging the system. The motherboard was controlled using a National Instruments' acquisition system, and two external battery power supplies were utilized for biasing the ROIC and generating the voltage levels needed for the operation of the TFTs.

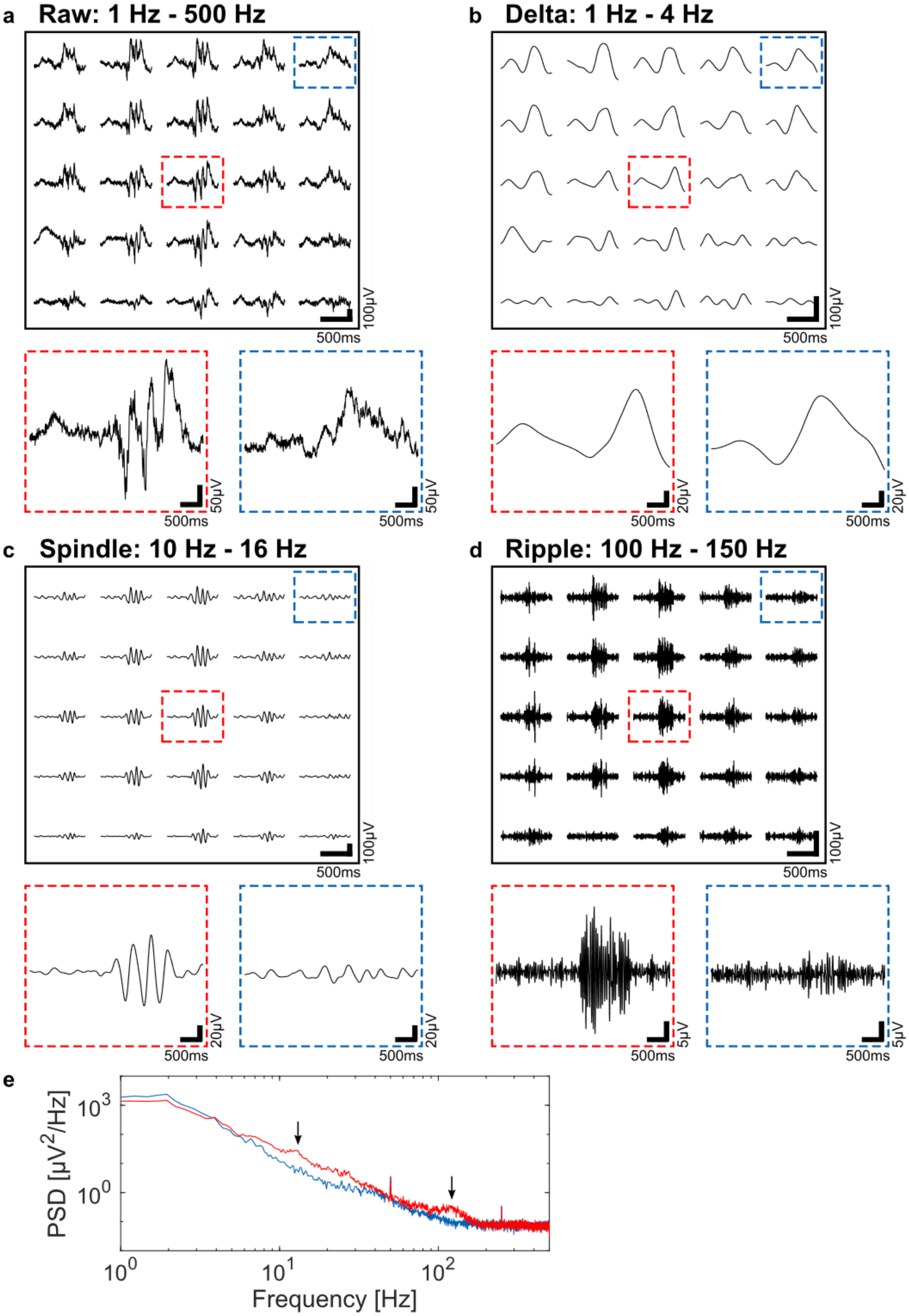
a
Raw: 1 Hz - 500 Hz
100µV
500ms
50µV
500ms
50µV
500ms
b
Delta: 1 Hz - 4 Hz
100µV
500ms
20µV
500ms
20µV
500ms
c
Spindle: 10 Hz - 16 Hz
100µV
500ms
20µV
500ms
20µV
500ms
d
Ripple: 100 Hz - 150 Hz
100µV
500ms
5µV
500ms
5µV
500ms
e
PSD [µV²/Hz]
10³
10⁰
10⁰
10¹
10²
Frequency [Hz]

Figure S14. Physiological oscillations under anesthesia for a subset of electrodes. Time traces of a) raw (1 Hz - 500 Hz) and b) to d) filtered signals for a subset of electrodes capturing high-frequency oscillations. Signals filtered in the b) delta (1 Hz - 4 Hz), c) spindle (10 Hz - 16 Hz), and d) ripple (100 Hz - 150 Hz) bands. For each frequency band, magnification of two representative electrodes is included, one capturing high-frequency oscillation (red dashed box) and the other without (blue dashed box). e) Power spectral densities for selected electrodes. The local peaks in the spindle band (~13 Hz) and ripple band (~120 Hz) present in one of the electrodes are indicated with arrows.

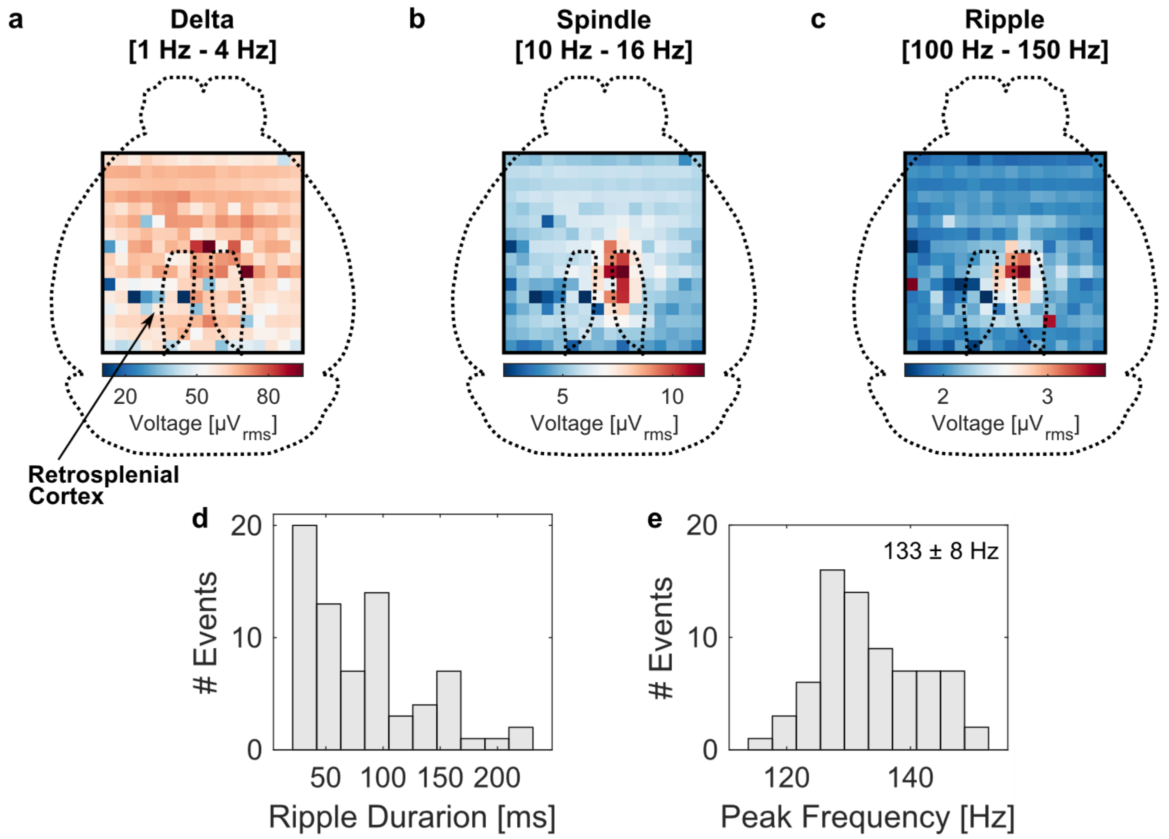

Figure S15. Color maps representing the RMS instantaneous power of spontaneous activity in the a) delta (1 Hz – 4 Hz), b) spindle (10 Hz – 16 Hz), and c) ripple (100 Hz – 150 Hz) bands. Localized spindle and ripple activity are seen over midline cortical structures. d) Histogram of ripple duration and e) ripple peak frequency, for a selected electrode presenting the highest power in the ripple band (n = 97 ripples from 129 s of recording in 1 mouse).

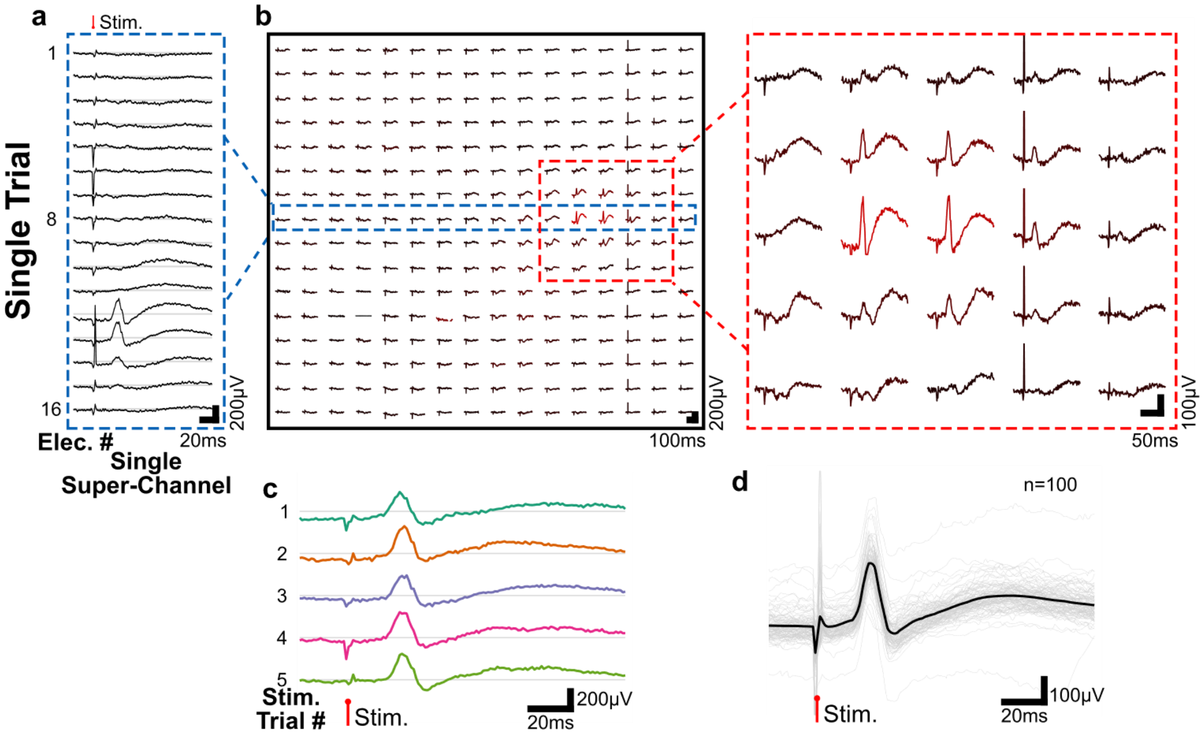

Figure S16. Single-trial response of somatosensory evoked potentials recorded using a μECoG array in time-division multiplexing mode. a) Response of 16 multiplexed electrodes sharing a single recording super-channel, and b) of all the 256 electrodes of the multiplexed array, showing the spatial distribution of the recorded evoked potential. A magnification of the electrodes around the elicited response is included. c) Difference in the evoked response after successive stimulation trials for a selected electrode. d) Averaged response of a selected electrode (gray: single trials, black: average value, n = 100).

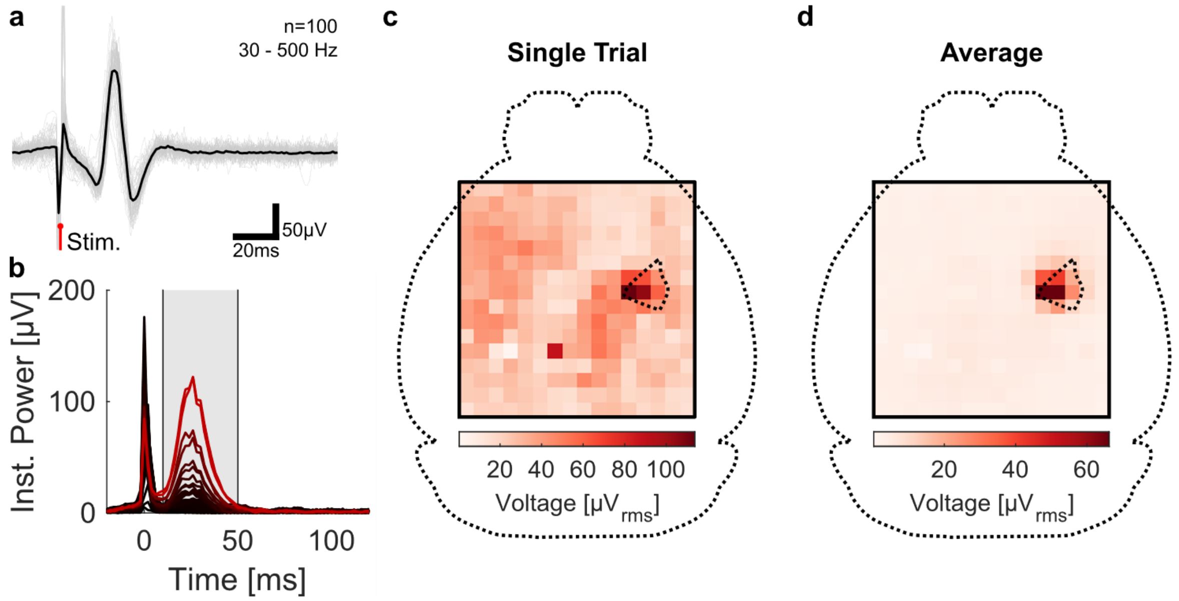

Figure S17. Localized gamma responses to electrical stimulation recorded with a 16×16 actively multiplexed array. a) Average gamma response of a selected multiplexed electrode recording SSEPs after electrical stimulation of one of the paws (gray: single trials, black: average value, n = 100). Trials aligned to the stimulation onset (TTL signal). b) Instantaneous power of the averaged responses filtered between 30 Hz and 500 Hz for all electrodes. The time frame used to calculate the RMS instantaneous power (10 to 50 ms after the stimulation onset) is highlighted. c) Single trial and d) average localized gamma response to electrical stimulation of the left hind paw (n = 100). The color map indicates the RMS instantaneous power in the specified time frame.

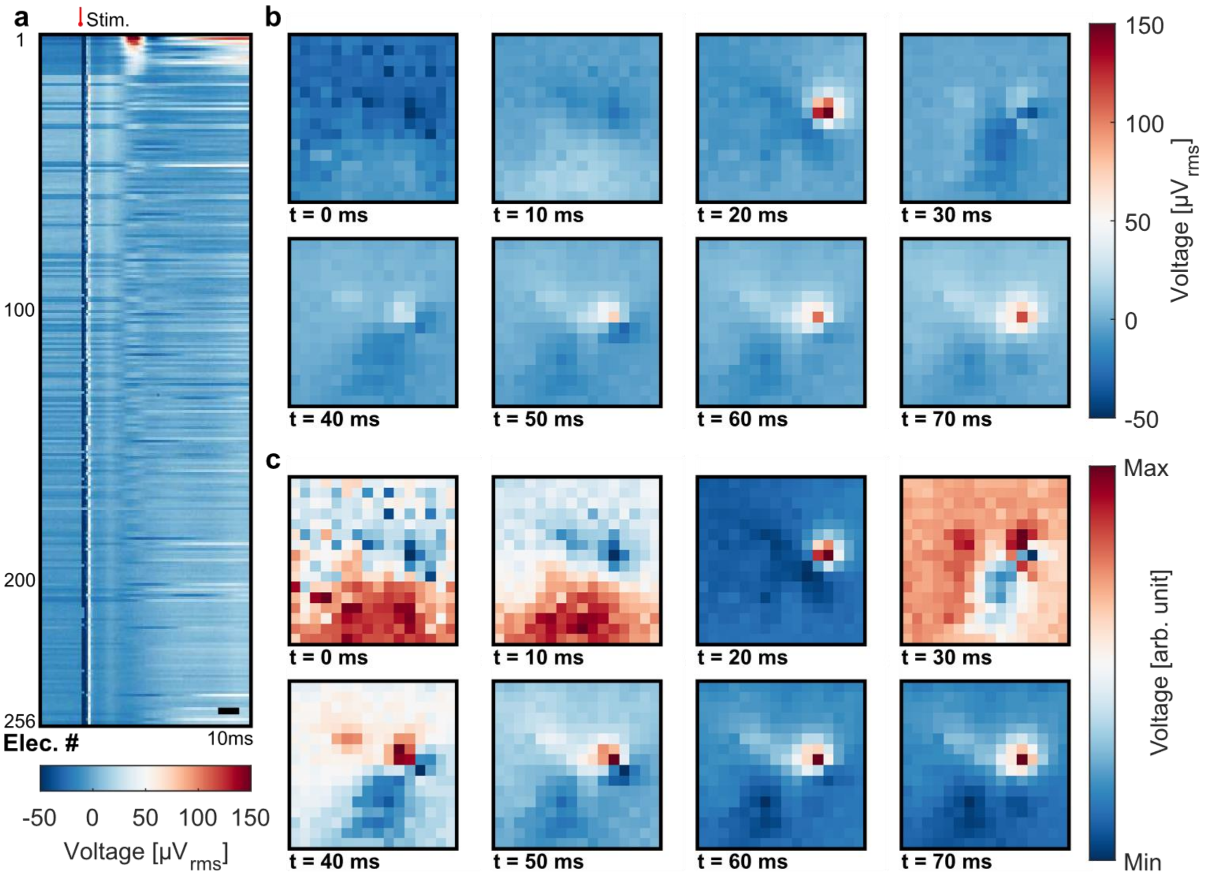

Figure S18. Spatiotemporal dynamics of averaged SSEPs (n = 100) for the first tens of milliseconds (75 ms) after electrical stimulation of the left hind paw. a) Color map of the averaged response for all 256 electrodes. b) Colormap sequences over time preserving the spatial organization of the array, where all frames share the range of the colorbar, and c) where each frame has its own range. For frames in b) and c), the pixels' color indicates the mean voltage calculated in 5 ms time bins.

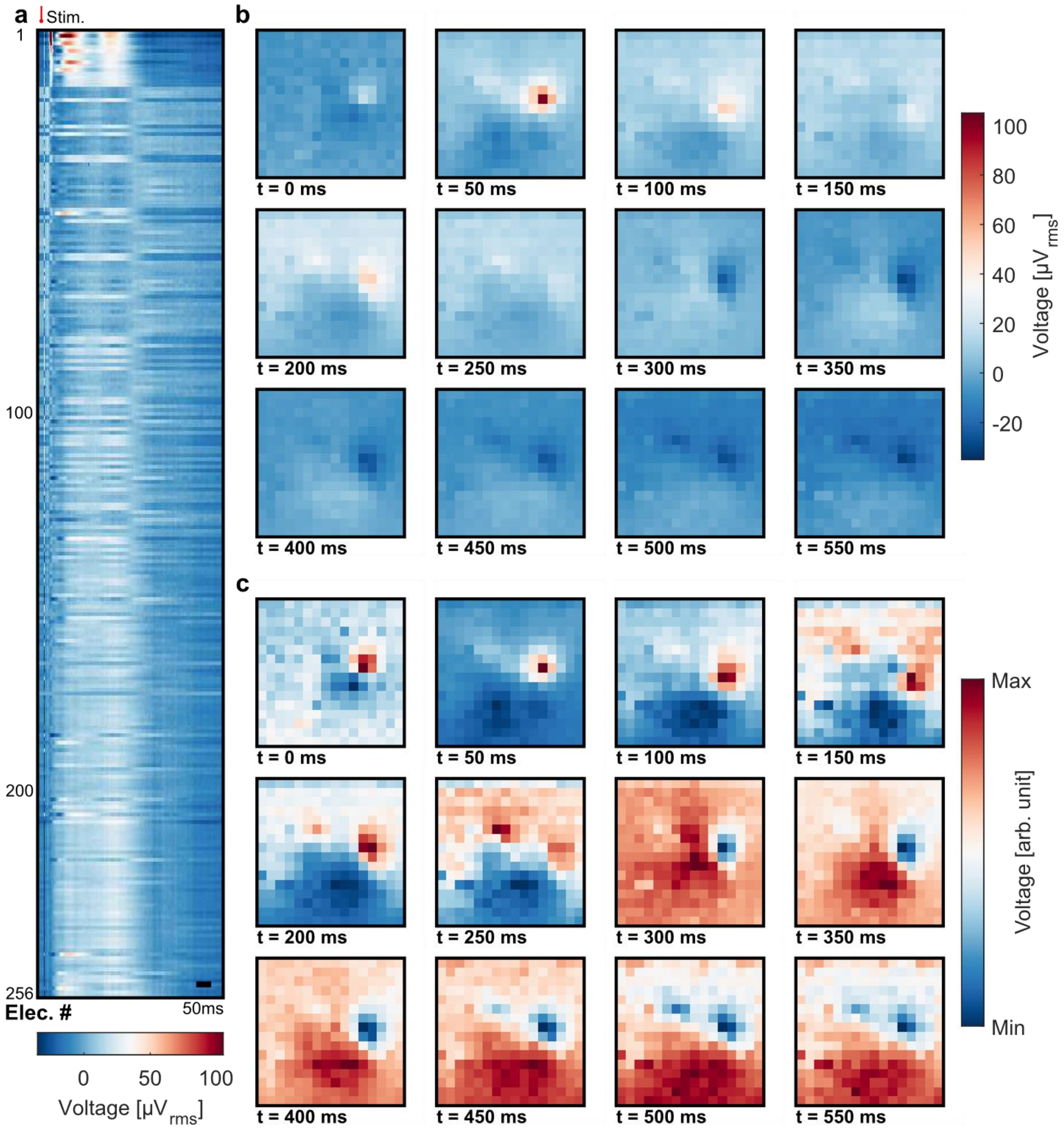

Figure S19. Spatiotemporal dynamics of averaged SSEPs (n = 100) for the first hundreds of milliseconds (600 ms) after electrical stimulation of the left hind paw. a) Color map of averaged response for all the 256 electrodes. b) Color map sequences over time preserving the spatial organization of the array, where all frames share the range of the colorbar, and c) where each frame has its own range. For frames in b) and c), the pixels' color indicates the mean voltage calculated in 50 ms time bins.

References – Supporting Information

[1] S. S. Stensaas, L. J. Stensaas, *Acta Neuropathol.* **1978**, *41*, 145.

[2] H. Charkhkar, C. Frewin, M. Nezafati, G. L. Knaack, N. Peixoto, S. E. Saddow, J. J. Pancrazio, *Biosens. Bioelectron.* **2014**, *53*, 316.

[3] G. Voskerician, M. S. Shive, R. S. Shawgo, H. von Recum, J. M. Anderson, M. J. Cima, R. Langer, *Biomaterials* **2003**, *24*, 1959.

[4] S. Kang, R. K. J. Murphy, S. Hwang, S. M. Lee, D. V. Harburg, N. A. Krueger, J. Shin, P. Gamble, H. Cheng, S. Yu, Z. Liu, J. G. McCall, M. Stephen, H. Ying, J. Kim, G. Park, R. C. Webb, C. H. Lee, S. Chung, D. S. Wie, A. D. Gujar, B. Vemulapalli, A. H. Kim, K.-M. Lee, J. Cheng, Y. Huang, S. H. Lee, P. V. Braun, W. Z. Ray, J. A. Rogers, *Nature* **2016**, *530*, 71.

[5] M. Caicedo, J. J. Jacobs, A. Reddy, N. J. Hallab, *J. Biomed. Mater. Res. Part A* **2008**, *86A*, 905.

[6] Y. Li, C. Wong, J. Xiong, P. Hodgson, C. Wen, *J. Dent. Res.* **2010**, *89*, 493.

[7] G. Kotzar, M. Freas, P. Abel, A. Fleischman, S. Roy, C. Zorman, J. M. Moran, J. Melzak, *Biomaterials* **2002**, *23*, 2737.

[8] C. Exley, E. R. House, *Monatshefte für Chemie - Chem. Mon.* **2011**, *142*, 357.

[9] T. Stieglitz, *J. Neural Eng.* **2009**, *6*, 065005.

[10] T. Stieglitz, H. Beutel, M. Schuettler, J. U. Meyer, È. Beutel, M. Schuettler, T. Stieglitz, J. U. Meyer, *Biomed. Microdevices* **2000**, *2*, 283.

[11] T.-M. Fu, G. Hong, T. Zhou, T. G. Schuhmann, R. D. Viveros, C. M. Lieber, *Nat. Methods* **2016**, *13*, 875.

[12] D. S. Finch, T. Oreskovic, K. Ramadurai, C. F. Herrmann, S. M. George, R. L. Mahajan, *J. Biomed. Mater. Res. Part A* **2008**, *87A*, 100.

[13] T. V Thamaraiselvi, S. Rajeswari, *Trends Biomater. Artif. Organs*, **2004**, *18*, 9.

[14] D.-H. Kang, J.-G. Choi, W.-J. Lee, D. Heo, S. Wang, S. Park, M.-H. Yoon, *APL Bioeng.* **2023**, *7*, 026102.

[15] M. Mierzejewski, H. Steins, P. Kshirsagar, P. D. Jones, *J. Neural Eng.* **2020**, *17*, 052001.

[16] X. Huang, H. Londono-Ramirez, M. Ballini, C. Van Hoof, J. Genoe, S. Haesler, G. Gielen, N. Van Helleputte, C. M. Lopez, in *2022 IEEE Int. Solid- State Circuits Conf.*,

IEEE, **2022**, pp. 200–202.

[17] X. Huang, H. Londoño-Ramírez, M. Ballini, C. Van Hoof, J. Genoe, S. Haesler, G. Gielen, N. Van Helleputte, C. M. Lopez, *IEEE J. Solid-State Circuits* **2022**, *57*, 3312.

Authors Contributions

	H. Londoño-Ramírez	X. Huang	J. Cools	A. Chrzanowska	C. Brunner	M. Ballini	L. Hoffman	S. Steudel	C. Rolin	C. Mora Lopez	J. Genoe	S. Haesler
Conceptualization	•						•					•
Microfabrication	•		•									
Recording electronics design		•				•				•		
In vitro characterization	•	•										
Surgery				•	•							
In vivo recordings	•	•		•								
Data analysis	•											
Writing: original draft	•											
Writing: review and editing	•	•	•		•	•	•	•		•	•	•
Supervision						•	•	•	•	•	•	•
